# Intron Definition and a Branch Site Adenosine at nt 385 Control RNA Splicing of HPV16 E6*I and E7 Expression

**DOI:** 10.1371/journal.pone.0046412

**Published:** 2012-10-04

**Authors:** Masahiko Ajiro, Rong Jia, Lifang Zhang, Xuefeng Liu, Zhi-Ming Zheng

**Affiliations:** Tumor Virus RNA Biology Section, HIV and AIDS Malignancy Branch, Center for Cancer Research, NCI, NIH, Bethesda, Maryland, United States of America; University of Kansas Medical Center, United States of America

## Abstract

HPV16 E6 and E7, two viral oncogenes, are expressed from a single bicistronic pre-mRNA. In this report, we provide the evidence that the bicistronic pre-mRNA intron 1 contains three 5′ splice sites (5′ ss) and three 3′ splice sites (3′ ss) normally used in HPV16^+^ cervical cancer and its derived cell lines. The choice of two novel alternative 5′ ss (nt 221 5′ ss and nt 191 5′ ss) produces two novel isoforms of E6E7 mRNAs (E6*V and E6*VI). The nt 226 5′ ss and nt 409 3′ ss is preferentially selected over the other splice sites crossing over the intron to excise a minimal length of the intron in RNA splicing. We identified AACAAAC as the preferred branch point sequence (BPS) and an adenosine at nt 385 (underlined) in the BPS as a branch site to dictate the selection of the nt 409 3′ ss for E6*I splicing and E7 expression. Introduction of point mutations into the mapped BPS led to reduced U2 binding to the BPS and thereby inhibition of the second step of E6E7 splicing at the nt 409 3′ ss. Importantly, the E6E7 bicistronic RNA with a mutant BPS and inefficient splicing makes little or no E7 and the resulted E6 with mutations of ^91^QYNK^94^ to ^91^PSFW^94^ displays attenuate activity on p53 degradation. Together, our data provide structural basis of the E6E7 intron 1 for better understanding of how viral E6 and E7 expression is regulated by alternative RNA splicing. This study elucidates for the first time a mapped branch point in HPV16 genome involved in viral oncogene expression.

## Introduction

Human Papillomaviruses (HPV) are a large group of small, non-enveloped, double stranded DNA tumor viruses, with a total of ∼120 genotypes [1]. A subset of them have been characterized to cause benign warts (low-risk HPVs) and human cancers (high-risk HPVs) [2], [3]. Persistent infection with high-risk HPVs is responsible for the development of more than 95% of cervical cancer, 50–90% of other anogenital cancers and 20–30% of oral and pharyngeal cancers [3]–[5]. Amongst high-risk HPVs, HPV16 is the most prevalent genotype responsible for ∼60% of cervical cancer [4], [6]. Viral E6 and E7 of high-risk HPVs are viral oncoproteins and respectively degrade p53 and pRB, two cellular tumor suppressors, thereby contributing to HPV carcinogenesis [2].

The HPV16 E6 and E7 genes are positioned side by side at the beginning of the papillomavirus genome and are initially transcribed as a single bicistronic pre-mRNA from a major early promoter P97. This bicistronic E6E7 pre-mRNA contains three exons and two introns and its intron 1 extends from E6 open reading frame (ORF) to E7 ORF and contains three alternative 3′ splice sites (3′ ss) [7]. Alternative splicing of the HPV16 intron 1 leads to production of three species of E6 mRNAs: E6*I (nt 226∧nt 409) and E6*II (nt 226∧nt 526) to disrupt the E6 ORF and an E6∧E7 (nt 226∧nt 742) to disrupt both E6 ORF and E7 ORF [8]–[10]. While the HPV18 E6*I mRNA translates a truncated E6 polypeptide [11], [12], the E6*I mRNAs of both HPV16 and HPV18 mainly function as an E7 mRNA to translate E7 protein [8], [13]. Because the E7 ORF is too close to the upstream E6 ORF in an unspliced E6E7 bicistronic transcript, this structure position of the viral E6 and E7 ORFs in an unspliced RNA prevents a scanning ribosome from reinitiating next run of E7 translation after termination of E6 translation [13].

However, a small proportion of the E6E7 bicistronic RNA with intron 1 retention is detectable in cervical cancer tissues and cervical cancer-derived cell lines and this species of E6E7 mRNA is responsible for translation of a full-length E6. Mechanistically, there are only limited data currently available on how the intron 1 escapes recognition by cellular splicing machinery to produce a full-length E6 protein. The cap-binding complex at the RNA 5′ end was initially found to promote splicing of the intron 1 of HPV16 E6E7 transcripts, but the cap-enhanced splicing of intron 1 can be restrained by the distance of intron 1 from its RNA 5′ cap [8]. Under natural conditions, epithelial growth factor (EGF) appears to regulate the intron 1 splicing of HPV16 E6E7 pre-mRNA via Erk1/2 activation [14]. It is also possible that the oncogenic HPVs retain an intron by using their own proteins to interfere with the splicing machinery. The findings of that both HPV16 E2 and E6 are RNA-binding proteins and suppress RNA splicing [15] and that viral E5 regulates EGF receptor expression [16] may help us to understand this striking phenomenon. However, none of these studies addressed how EGF or viral proteins inhibit the assembly of the RNA splicing machinery on the intron 1. To address this question, we decided to characterize the HPV16 intron 1 and it′s BPS which could be essential for understanding this sophisticated regulation of E6E7 RNA splicing.

In this report, we have carefully characterized the intron 1 of HPV16 E6E7 pre-mRNA and identified two novel 5′ splice sites (5′ ss) and a functional BPS that controls selection of the intron 1 nt 409 3′ ss for production of E6*I. Our data indicate that splicing of the HPV16 intron 1 is coordinated with a simple principle of proximal selection over the intron for preferential excision of a minimal length of the intron.

## Materials and Methods

### Cell Lines

CaSki and SiHa cells are cervical cancer cell lines with integrated HPV16 genome. W12 cell line was originated from a CIN I lesion [17] and its subclone cell lines of 20861 and 20863 contain, respectively, an integrated and episomal HPV genome [18]. Green monkey kidney CV-1 cells, human osteosarcoma U2OS cells, human embryonic kidney HEK293 cells and human colorectal carcinoma HCT116 cells were purchased from American Type Culture Collection (Manassas, VA). 20861 and 20863 cells were grown on J2 feeder cells in the presence of F medium as described [18]. All other cell lines were grown at 37°C in the presence of 5% CO_2_ in Dulbecco′s modified Eagle′s medium (Invitrogen, Carlsbad, CA), except U2OS and HCT116 cells in McCoy′s 5a medium (Invitrogen), supplemented with 10% fetal bovine serum (HyClone, Logan, UT), 2 mM L-glutamine, 100 U/ml penicillin, and 100 µg/ml streptomycin.

### Plasmids

Plasmid pSB22 was constructed to express HPV16 E6 ORF (nt 104–586) with a disrupted nt 226 5′ ss (GU to GG mutation or U228G mutation) driven by a cytomegalovirus immediate early (CMV IE) promoter. The pSB22 was derived from plasmid pTMF1 [8] by swapping a polymerase chain reaction (PCR) product of pTMF1 amplified with a primer set of oSB76 and oSB77 into a pEGFP-C1 vector (Clontech, Mountain View, CA) at BamHI and HindIII sites. Plasmid pMA16 was constructed to express HPV16 E6E7 from nt 81–885 under control of the CMV IE promoter by insertion of a PCR product of HPV16 genomic DNA using a primer set of oZMZ209 and oZMZ262 into a pEGFP-N1 vector (Clontech) at Asp718 and XhoI sites. A series of HPV16 E6E7 (nt 81–880) expression vectors with a mutant (mt) BPS were constructed from pMA16 by PCR-based mutagenesis as described [19]. These includes pMA5 (mt-1), pMA6 (mt-2), pMA2 (mt-3), pMA3 (mt-4), pMA1 (mt-5), pMA17 (mt-6), pMA18 (mt-7), pMA19 (mt-12, G328C), pMA26 (mt-8), pMA27 (mt-9), pMA28 (mt-10), and pMA29 (mt-11). See details in Table S1 and Table S2 for each plasmid construction strategy and the primer sets used for the construction by PCR-based mutagenesis with an overlapping PCR technique [19]. All wt and mt expression vectors were verified by sequencing.

### Western Blotting

HCT116 cells were transfected with pMA16, pMA18, pMA26 or p3xFLAG CMV-14 (Sigma-Aldrich, St. Louis, MO) and were collected 48 h after transfection. Cell lysates were then analyzed for protein expression with anti-p53 (DO1) mouse monoclonal antibody (Merck KGaA, Darmstadt, Germany), anti-HPV16 E7 (ED17) mouse monoclonal antibody (Santa Cruz Biotechnology, Santa Cruz, CA) and anti-β-actin (Ac-15) mouse monoclonal antibody (Santa Cruz Biotechnology).

### Computational Analysis for 5′ ss, 3′ ss and BPS

5′ ss and 3′ ss matrix score were calculated by using ESE finder software (http://rulai.cshl.edu/ESE) [20], [21], based on the sequence similarity of 30 nucleotides surrounding a potential splice site to that of 27,556 constitutive splicing sites in dbCASE (http://rulai.cshl.edu/dbCASE) [22]. Consensus value (CV) of BPS was calculated by using Human Splice Finder (http://www.umd.be/HSF/) [23], representing position weight matrix score based on the similarity to the experimentally validated 14 BPS [23].

### RNA Preparations and Reverse Transcription (RT)-PCR

HEK 293 cells (8×10^5^) or CV-1 cells (1.0×10^6^) in a 6-cm dish were transfected with 2 µg of pSB22 by Lipofectamine 2000 (Invitrogen) or HPV16 E6E7 expression vectors with LipoD293 transfection reagent (SignaGen, Rockville, MD). Total RNA at 24 h after transfection was extracted using TRIzol (Invitrogen) and treated by RNase-free DNase I to remove plasmid DNA. The same protocol was also used to prepare total RNA from CaSki, SiHa, and W12 subclone cells 20861 and 20863. Total RNA from cervical cancer (Ca) or normal (N) tissues pretreated with DNase I (Catalog number: 7277 [Ca 1], 7992[N1], 7276-T [Ca 2], and 7276-N [N2]) was purchased from Ambion (Austin, TX) or obtained as gifts (Ca 3 and Ca 4) from Yang Li and Xing Xie of Zhejiang University, China. Poly (A)-selected mRNA was prepared from total RNA by using QuickPrep mRNA Purification Kit (GE Healthcare, Little Chalfont, UK). For RT-PCR detection, total RNA (0.25 µg) was reverse transcribed by MultiScribe Reverse Transcriptase (Applied Biosystems, Foster City, CA) with random hexamer and then amplified by AmpliTaq (Applied Biosystems) in the presence of a specific primer set as described in each figure and detailed in Table S2. Signal intensity of each PCR product was quantified by Image Lab Software version 3.0 (Bio-Rad Laboratories, Hercules, CA). All RT-PCR products were confirmed by sequencing.

### *In vitro* Transcription

Templates used to transcribe HPV16 E6E7 pre-mRNA (nt 107–889 or nt 107–881 attached with a U1 binding site) were prepared from plasmid pMA16 to have a 5′ T7 promoter by PCR amplification using a primer set of oZMZ208 and oZMZ275 (Table S2) for a template from nt 107 to nt 889 or oZMZ208 and oZMZ276 (Table S2) for a template from nt 107 to nt 881 plus a consensus U1 binding site. Templates for HPV16 pre-mRNAs (nt 107–540) with wild type (wt) BPS, mt-7 BPS, and mt-11 BPS containing a 5′ T7 promoter and a 3′ U1 binding site were obtained by PCR amplification from pMA16, pMA18, and pMA29, respectively, with a primer set of oZMZ208 and oZMZ221 (Table S2). The PCR products (∼500 ng) were then used for *in vitro* transcription with Riboprobe System-T7 (Promega, Fitchburg, WI) in 50 µl of 1 X transcription buffer [10 mM DTT, 20 U RNase inhibitor, 500 µM rATP, 500 µM rUTP, 50 µM rGTP, 50 µM rCTP, 500 µM m7G(5′)ppp(5′) (New England Biolabs, Ipswich, MA), 80 µCi of [α-^32^P] rCTP and 80 U T7 RNA polymerase]. The *in vitro* transcription was carried out at 37°C for 2 h and the DNA template was removed with TURBO DNA-free enzyme (Invitrogen). The transcription products were resolved in a 6% denaturing PAGE gel containing 7.5 M Urea (National Diagnostics, Atlanta, GA), and the full length transcripts were cut and eluted from the PAGE gel and subsequently purified and quantified.

### *In vitro* RNA Splicing Assay

For *in vitro* splicing assay, 4.0 ng of [α-^32^P] rCTP-labeled HPV16 E6E7 pre-mRNA transcribed *in vitro* were applied for *in vitro* RNA splicing as described [24]. A 100-bp DNA Ladder (Invitrogen) labeled with [γ-^32^P] ATP was used as a size marker.

### U2 Depletion from HeLa Nuclear Extract

DNA oligo-directed U2 snRNA depletion was performed as described previously with a few modifications [8], [25], [26]. HeLa nuclear extract (8 µl, 7.5 µg of total protein/µl) was treated with 1.5 unit of RNase H (Promega) in the absence or presence of 1, 2, or 5 µM oMA92 (Table S2), an oligo DNA complimentary to the nt 22–36 of human U2 snRNA, for 30 min at 30°C to deplete U2 snRNA in a final reaction volume of 20 µl in the presence of a reaction buffer containing final concentration of 40% HeLa nuclear extract, 5.0 mM HEPES (pH 7.9), 0.6% polyvinyl alcohol, 0.4 mM ATP, 20 mM creatine phosphate, 3 mM MgCl2, 0.075 U/µl RNase H. The nuclear extract with or without U2 depletion was then verified by Northern blot and was used for RNase H protection assay.

### Northern Blot

U2 depletion efficiency of HeLa nuclear extract was examined by Northern blot. Briefly, total RNA from 8 µl of HeLa nuclear extract with or without U2 depletion was extracted by phenol/chloroform/isoamyl alcohol, dissolved in 10 µl of NorthernMax formaldehyde loading dye (Ambion), denatured at 75°C for 10 min, resolved in 6% denaturing PAGE gel containing 7.5 M Urea (National Diagnostics), and transferred onto a nylon membrane with a constant currency of 400 mA for 2 h. After prehybridization for 1 h at 42°C in Super-Hybridization buffer (Sigma), hybridization was carried out for 16 h at 42°C with an antisense DNA oligo oKY48 for U1 snRNA, oKY50 for U2 snRNA, or oST197 (Table S2) for U6 snRNA labeled with [γ-^32^P] ATP. The membrane was then washed once with a 2X SSPE (1X SSPE contains 180 mM NaCl, 10 mM NaH_2_PO_4_ and 1 mM EDTA, pH 7.7) −0.5% SDS for 5 min at 42°C, twice with 0.2X SSPE−0.1% SDS for 30 min at 42°C, and exposed to a phosphoimager screen.

### RNase H Protection Assay

Four ng of [α-^32^P] rCTP-labeled pre-mRNA transcribed *in vitro* was mixed with 8 µl (7.5 µg/µl) of HeLa nuclear extract in a reaction volume of 20 µl in the presence of the reaction buffer containing final concentration of 40% HeLa nuclear extract, 5.0 mM HEPES (pH 7.9), 0.6% polyvinyl alcohol, 0.4 mM ATP, 20 mM creatine phosphate, 3 mM MgCl2. After incubation at 30°C for 30 min, the mixture was incubated for additional 10 min at 30°C in the presence of 500 nM DNA oligo oMA64 for wild type BPS, oMA76 for mt-11 BPS, oMA60 for mt-7 BPS, or oMA61 (Table S2) for PPT, depending on which ^32^P-labeled pre-mRNA was used. Subsequently, the reaction was digested at 30°C for 10 min, terminated by addition of 60 µl of proteinase K solution (5 mg/ml proteinase K [Roche Diagnostics], 50 mM EDTA, 0.5% [W/V] SDS) and incubated for 20 min at 75°C. The digested products were extracted by phenol/chloroform/isoamyl alcohol and were resolved in a 6% denaturing PAGE gel containing 7.5 M Urea (National Diagnostics). The PAGE gel was then transferred onto a 3M filter paper and dried. The radioactivity of the digested products was captured by exposing the filter paper to a storage phosphor screen (Molecular Dynamics). The screen was then imaged by a PhosphoImager STORM 860 and analyzed with ImageQuant software (Molecular Dynamics).

HeLa nuclear extract with U2 depletion was also used for RNase H protection assays. In this case, a ^32^P-labeled pre-mRNA (4 ng) and a corresponding BPS-specific DNA oligo (oMA64 for wt BPS, oMA76 for mt-11 BPS) were added to each depletion reaction described above, and the mixture was incubated for 10 min at 30°C before proteinase K digestion [25].

## Results

### Identification of Two Novel Alternative 5′ ss in the HPV16 Intron 1

**Figure 1:**
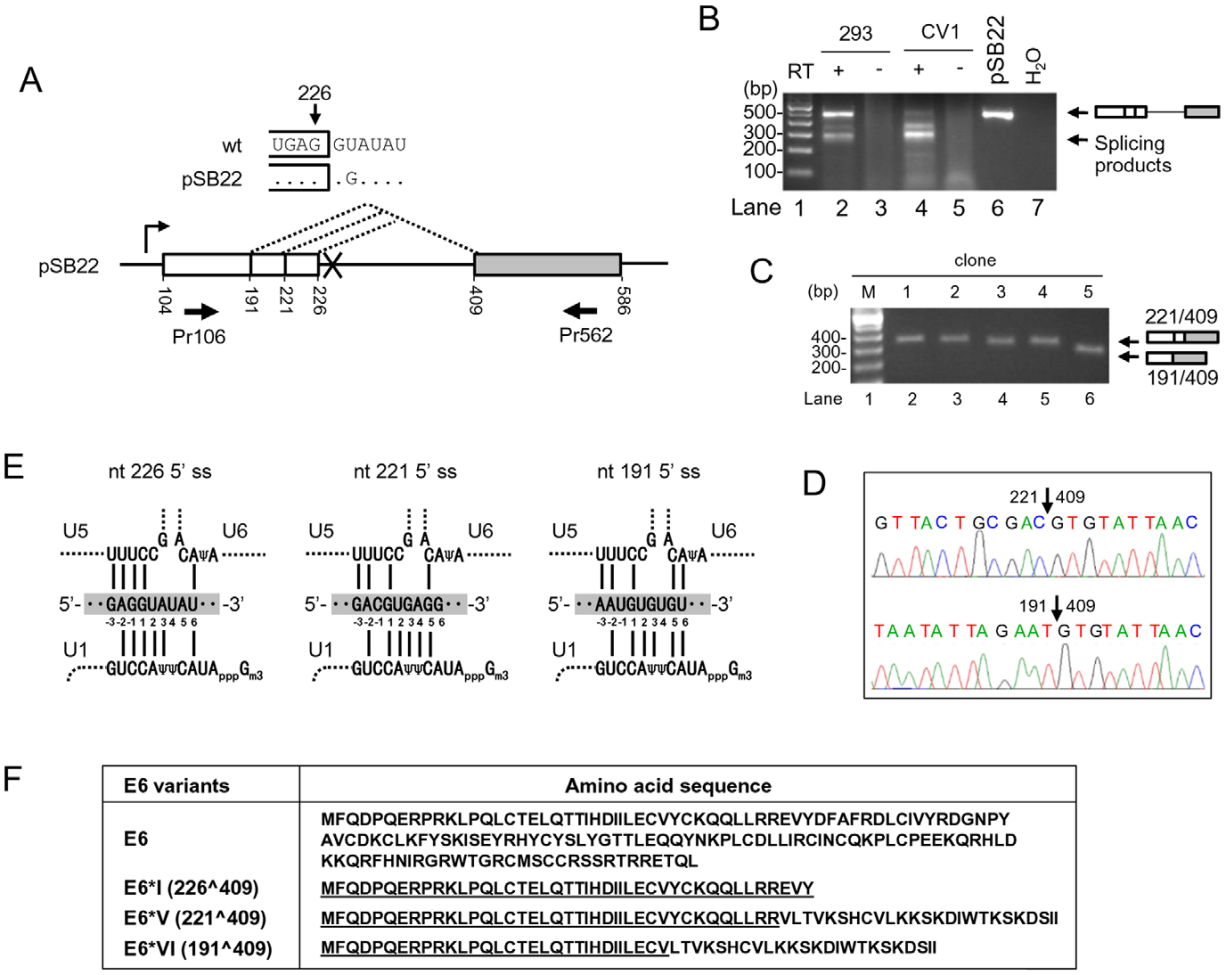
Identification of two novel alternative 5′ ss in the HPV16 intron 1. (A) Diagram of alternative 5′ ss identified in this study by using pSB22, an HPV16 E6 expression vector containing a mutated nt 226 5′ ss (GU to GG, U to G at nt 228). Numbers below the pre-mRNA indicate nucleotide positions in the HPV16 genome. Boxes, exons; lines, introns; dashed lines, splicing directions. Arrows below the exons are a pair of primers used for RT-PCR. (B) RT-PCR result of alternative RNA splicing of E6 pre-mRNA in HEK293 or CV1 cells transfected with pSB22. RT-PCR products with a size of ∼300 bp were gel-purified and cloned. (C) Plasmid DNAs extracted from five bacterial colonies were digested by EcoRI and analyzed by agarose gel electrophoresis, showing the insert size. (D) Sequence chromatograms showing splice junctions of colonies 1–4 (nt 221/409) and the colony 5 (nt 191/409). (E) Illustration of the base pairing of three alternative 5′ ss (shaded, middle) in HPV16 intron 1 with U1, U5 and U6 snRNAs. Exon sequences are labeled as −1, −2 and −3 from the 5′ end of the intron. Base pairing is indicated with solid lines. Ψ, pseudo-uridine; Gm3, 2,2,7-tri-methyl guanosine. (F) Coding potentials of spliced E6E7 transcripts by using the nt 221 (E6*V) or nt 191 (E6*VI) 5′ ss. Amino acid residues underlined in E6*I, E6*II, E6*V and E6*VI are identical to the N-terminus of wt E6.

To express full-length E6 ORF from a splicing defect construct, an E6 expression vector (pSB22) carrying a mutation at nt 226 5′ ss (GU to GG, U to G mutation at nt 228) (Fig. 1A) in the E6 coding region was initially used to transcribe full-length, unspliced E6 coding region in mammalian cells. Unexpectedly, we found that the mutant RNA was spliced efficiently in CV-1 cells and less efficiently in HEK293 cells (Fig. 1B). Cloning and sequencing of the spliced products in size of ∼300 bps from the mutant RNA revealed two novel 5′ ss, nt 221 5′ ss and nt 191 5′ ss, that were selected for the splicing of the mutant RNA. Based on their cloning frequency, usage of the two alternative 5′ ss was in a ratio of ∼4∶1 (nt 221: nt 191) (Fig. 1C–D). Sequence analysis of all three 5′ ss shows that they are all deviated from a consensus mammalian 5′ ss, yag/GURAGU, recognizable by U1 snRNP, and then replaceable by U5 snRNA and U6 snRNA at two catalytic steps of RNA splicing [27], [28]. All of three 5′ ss of nt 226, nt 221, and nt 191 show suboptimal match to the U1 snRNA binding site (Fig. 1E). There are two nucleotides at the nt 226 5′ ss intron positions 4 and 5 missing for base-pairing to the core nucleotides from the U1 5′ end. Similarly, a single nucleotide at the exon –1 position in both nt 221 and nt 191 5′ ss and an additional nucleotides at position 6 in the nt 221 5′ ss and at position 4 in the nt 191 5′ ss are missing for base-pairing to the U1 snRNA binding site (Fig. 1E). By using the ESEfinder (http://rulai.cshl.edu/ESE), we obtained a matrix score for each 5′ ss, with a strength order for nt ine226 (5.682) > nt 221 (4.338) > nt 191 (3.853) (Table S3). Moreover, we found that selection of an alternative 5′ ss at nt 221 or nt 191 for splicing to nt 409 3′ ss provides an ORF for a novel E6 isoform and we referred it to as E6*V for the nt 221 5′ss usage and E6*VI for the nt 191 5′ ss usage (Fig. 1F) after the names of E6*I-E6*IV [7]. Similar to E6*I and E6*II, the N-terminal halves of both E6*V and E6*VI are identical to the N-terminus of E6, but differ from E6 and E6*I in their C-termini. Whether E6*V and E6*VI could function as described for E6*I [11], [12] remains to be investigated.

### Intron Definition Promotes Selection of a Proximal 3′ ss and 5′ ss Over the HPV16 Intron 1 during Virus Infection

**Figure 2:**
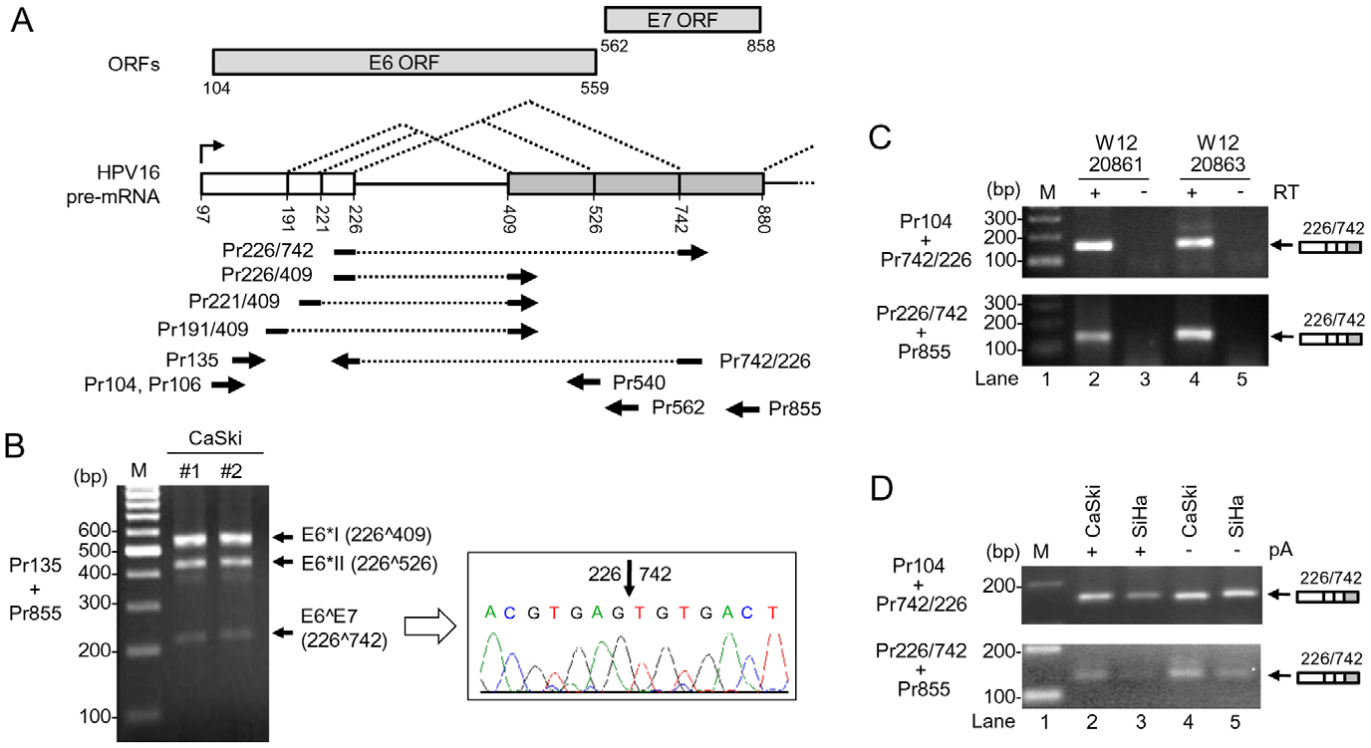
Utilization of nt 742 3′ ss in multiple HPV16^+^ cervical cancer cell lines. (A) Diagram of splicing directions of HPV16 E6E7 pre-mRNA from three alternative 5′ ss at nt 191, 221, and 226 to three alternative 3′ ss at nt 409, 526 and 742. Numbers represent nucleotide positions in the HPV16 genome. Arrows under the E6E7 pre-mRNA structure indicate primers used in RT-PCR in this and all other figures. Rectangles above the E6E7 pre-mRNA structure indicate ORFs of E6 and E7. (B) Two preps of total RNA extracted from CaSki cells were analyzed by RT-PCR with a primer pair of Pr135 and Pr855 (left). The 214 bp RT-PCR product was purified from the gel and sequenced as a splicing product from nt 226 to 742 (right). (C) E6∧E7 expression is detectable by RT-PCR from W12 subclone cell lines 20861 and 20863 which harbor integrated and episomal HPV16 genome, respectively. (D) E6∧E7 expressed in HPV16^+^ CaSki and HPV16^+^ SiHa cells are polyadenylated. Total RNA with or without polyadenylation (pA) was used for RT-PCR with the indicated splice junction primers on the left (C and D).

**Figure 3:**
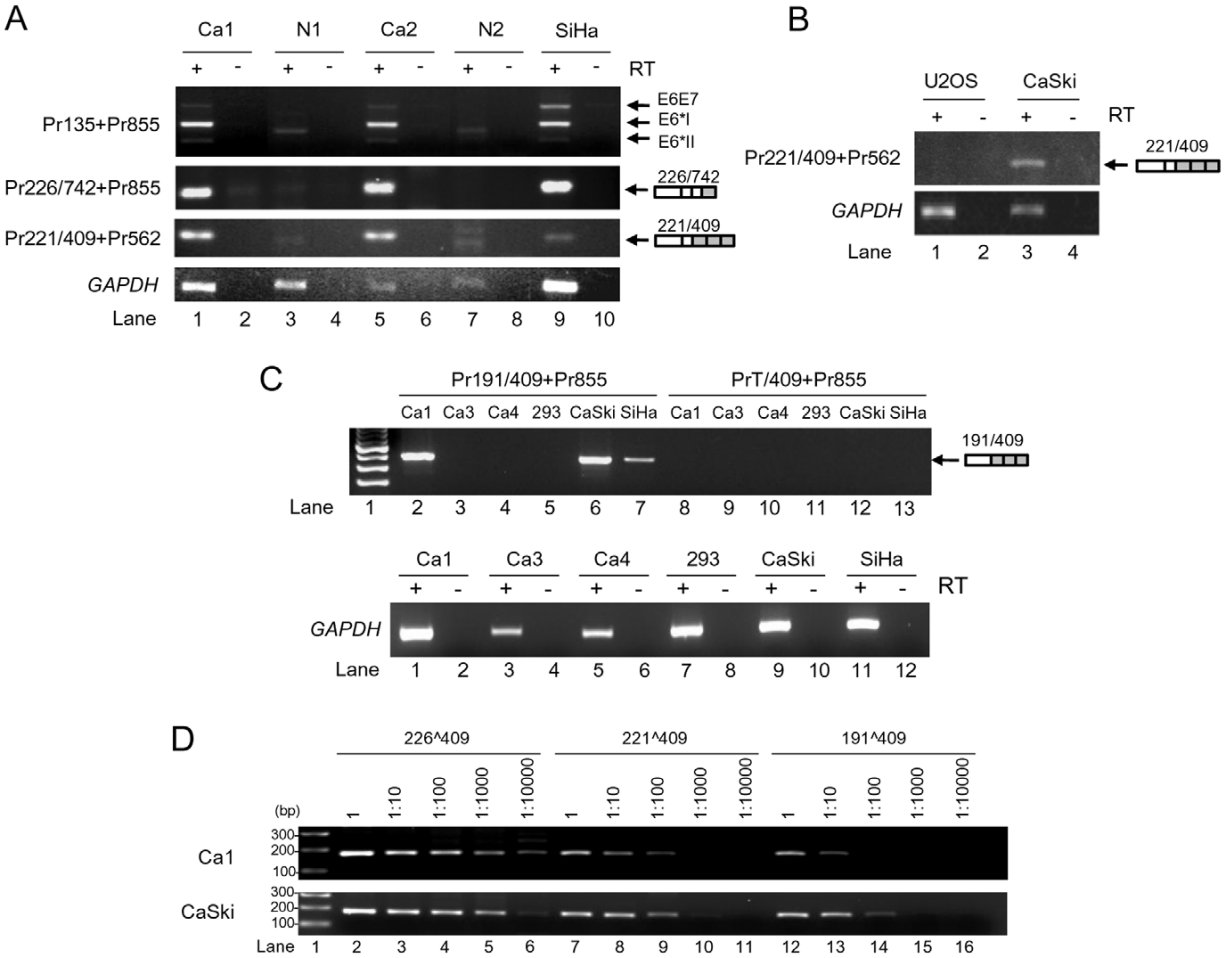
Usage of alternative 5′ ss and 3′ ss in HPV16^+^ cervical cancer tissues and cell lines. (A) RT-PCR for total RNA from cervical tissues and SiHa cells with the indicated primer pairs (Fig. 2A) on the left, showing HPV16 splicing product from nt 221 5′ ss to nt 409 3′ ss in HPV16^+^ cervical cancer tissues (Ca) and SiHa cells. Expression of E6∧E7, E6*I, and E6*II served as positive controls. Normal cervical tissues (N) served as negative controls. A relative smaller or larger band in the normal tissue was a non-specific amplicon. (B) RT-PCR results for total RNA from HPV16^+^ CaSki cells and U2OS cells for splicing product from nt 221 to nt 409. HPV16-negative U2OS cells served as a negative control. (C) RT-PCR results of total RNA from HPV16^+^ cervical cancer tissues, CaSki, SiHa and HEK293 cells, showing the splicing product from nt 191 to nt 409. A 191∧409 splice junction 5′ primer and its 3′ half primer starting from nt 409 3′ ss were used in combination with a 3′ primer Pr855 for this comparison. HPV16-negative HEK293 RNA served as a negative control. GAPDH RNA served as a loading control. (D) Titration of the relative usage of nt 226, 221 and 191 5′ ss in cervical cancer tissues and CaSki cells by RT-PCR. The cDNAs of Ca1 and CaSki from panel C were serially diluted and amplified for the splicing products of 226∧409, 221∧409 and 191∧409 with each corresponding splice junction 5′ primer Pr226/409, Pr221/409, or Pr191/409 in combination of a common 3′ primer Pr562.

It has been well documented that HPV16 E6E7 pre-mRNA is predominantly spliced from nt 226 5′ ss to nt 409 3′ ss to make an E6*I transcript or to nt 526 3′ ss to make an E6*II transcript. We and others had also identified the third 3′ ss at nt 742 in the HPV16 genome to encode a E6∧E7 fusion protein [8]–[10]. However, a comparative analysis of the nt 742 3′ ss usage in HPV16^+^ cervical cancer cell lines has not been carefully studied. Data in Fig. 2A–D indicate that the identified nt 742 3′ ss as an alternative 3′ ss is detectable in cervical cancer-derived cell lines CaSki (∼500 copies of HPV16 genome), SiHa (1–2 copies of HPV16 genome), and W12 subclones 20861 (∼30 copies of integrated HPV16 genome) and 20863 (∼1000 copies of episomal HPV16 genome) cells [18] and accepts the nt 226 5′ ss for RNA splicing to encode the hypothetical E6∧E7 fusion protein [7]. The RNA spliced at the nt 742 3′ ss in CaSki and SiHa cells could be detected from total RNA or poly(A)-selected RNA preps (Fig. 2D). When compared with two other well-described 3′ ss, nt 409 and nt 526, the nt 742 3′ ss was utilized much less in CaSki cells (Fig. 2B). Sequence analysis showed a weak polypyrimidine tract (PPT) interspersed by Gs or As and a putative suboptimal BPS in all three 3′ ss. By using ESEfinder, we obtained a comparative strength of matrix score for each 3′ ss in the order of nt 526 3′ ss (9.130) > nt 409 3′ ss (2.563) > nt 742 3′ ss (1.624) (Table S3). The finding of that the nt 409 3′ ss has a matrix score weaker than the nt 526 was unexpected because the nt 409 3′ ss is preferentially selected for splicing of the E6E7 intron 1 in cervical cancer tissues and and their derived cell lines (Fig. 2B and 3A). Data suggest that intron definition favors a proximal 3′ ss over a distal 3′ ss in the RNA splicing.

Intron definition indicates that pairing of splice sites in eukaryotes most likely occurs across introns in a manner that favors excision of the smallest segment [29]–[31] and has been efficiently used by minute virus of mice [32]. To examine whether the newly identified 5′ ss is normally utilized during HPV16 infection, we examined two pairs of total RNA from normal cervix and cervical cancer by RT-PCR with a specific splice-junction primer (Fig. 2A). Normal cervix and HPV16-negative U2OS cells served as a negative control. Consistent with previous reports [8]–[10], [13], data in Fig. 3A show that both the nt 226 5′ ss and the nt 409 3′ ss are preferentially selected over the other splice sites in cervical cancer and its derived cell lines for the E6*I splicing (see the result from primer set Pr135+Pr855) (top panel, lanes 1, 5 and 9). These could be a result of that splicing of HPV16 intron 1 5′ ss presumably takes place by intron definition and a proximal 5′ ss is favored cross over the intron. We noticed that the transcripts derived from usage of the nt 221 or 191 5′ ss were undetectable because of their less abundance in CaSki and SiHa cells and in cervical cancer tissues, when a PCR primer set (Pr135 and Pr855) derived from the regions upstream and dowmstream of splice sites which selectively amplifies a relative abundant viral transcript was used in RT-PCR assays (Fig. 2B and 3A).

When a splice junction-specific primer set of Pr221/409 and Pr855 was used in our RT-PCR assays, however, we could easily detect and confirm by sequencing the nt 221 5′ ss usage for splicing of the E6E7 bicistronic RNA in HPV16-positive cervical cancer tissues and cervical cancer cell lines, SiHa and CaSki cells (Fig. 3A, lanes 1, 5 and 9; Fig. 3B, lane 3; Fig. S1), but not in U2OS cells (Fig. 3B, lane 1) or normal tissues where only a very weak, non-specific band in the smaller or larger size was seen (Fig. 3A). As expected, RNA splicing from nt 226 to nt 742 (E6∧E7) was detected in the cancer tissues or SiHa cells with HPV16 infection (Fig. 3A, lanes 1, 5, and 9 in the top second panel) by a splice junction-specific primer pair of Pr226/742 and Pr855 (Fig. 2A). RNA splicing from nt 191 to nt 409 (Fig. 3C, lanes 2, 6 and 7) was also detected in one of cervical cancer tissues and two tested cervical cancer cell lines, CaSki and SiHa, by RT-PCR using another splice junction-specific primer set of Pr191/409 and Pr855 (Fig. 2A) and verified by sequencing (Fig. S1), but was not so by its 3′ half primer plus Pr855 (Fig. 3C, lanes 8–13). Together, our data indicate that the identified nt 221 5′ ss and nt 191 5′ ss are not crytic and are normally utilized during HPV16 infection. To determine the relative level of each 5′ ss usage in splicing of HPV16 E6E7 intron 1 in cervical cancer and its derived cell lines, we titrated the level of each spliced transcript by serial dilution in our semi-quantitative RT-PCR with each splice junction-specific primer set. We found that the level of 226∧409 transcripts both in cervical cancer tissue Ca1 and in CaSki cells was ∼100 times higher than the 221∧409 transcripts or ∼1000 times higher than the 191∧409 transcripts (Fig. 3D). Thus, our data clearly indicate that the selection of alternative 5′ ss in the HPV16 E6E7 intron 1 also follows the proximal rule as seen in the selection of alternative 3′ ss.

### Selection of the nt 409 3′ ss in Splicing of the HPV16 Intron 1 is Not Defined by Exon Definition

As E1 ORF spans over the entire intron 2 of viral early transcript, retention of the intron 2 is essential for E1 production. Our previous analysis of the intron 2 5′ ss, nt 880 5′ ss, indicated that two nucleotides at the intron positions 4 and 5 for base-pairing to the six core nucleotides from the U1 5′ end are missing, defining the nt 880 5′ ss as a weak 5′ ss [8], presumably leading to the intron 2 retention. The matrix score analysis of the nt 880 5′ ss showed a score of 5.132 (Table S3), relatively less than a score for the nt 226 5′ ss (5.682). The weak feature of the nt 880 5′ ss was assumed to be incapable for stimulating the selection of an upstream 3′ ss because exon definition relies on cross-talk between a 3′ ss and a 5′ ss over the exon. An oversized exon (>500 nts) also prevents such cross-talk and recognition of an upstream 3′ ss by the cellular machinery [33], [34]. As HPV16 E6E7 pre-mRNA primarily selects the intron 1 nt 409 3′ ss for RNA splicing in cervical cancer and its derived cell lines, this 3′ ss selection makes the RNA exon 2 in size of 477 nts, close to the size limit of exon definition [34]. This suggests why the selection of the nt 409 3′ ss for RNA splicing, as shown in Fig. 2B and Fig. 3A, is preferentially determined by intron definition, rather than exon definition. To test this possibility, HPV16 E6E7 transcripts with a native (nt 880 5′ ss) or consensus U1 binding site were synthesize by *in intro* transcription and compared for *in vitro* RNA splicing (Fig. S2A and S2B). We found that the RNA with a consensus U1 binding site (pre-mRNA 2) spliced efficiently at nt 742 3′ ss, whereas the RNA with a native U1 binding site (nt 880 5′ ss, pre-mRNA 1) did not (Fig. S2C and S2D). It is worth noting that the first step of RNA splicing at nt 226 5′ ss in the pre-mRNA 1 appeared normal by production of a splicing intermediate containing a lariat structure with slow migration in electrophoresis (Fig. S2C, lane 4), but its second step of RNA splicing at an alternative 3′ ss was blocked by an inefficient exon definition, thereby preventing the production of a fully spliced RNA (Fig. S2C, compare lanes 4 to 5). Data indicate that a weak nt 880 5′ ss in HPV16 E6E7 bicistronic RNA conferred the ability for the virus preferentially utilizing the intron definition over the exon definition to select the nt 409 3′ ss in the intron 1 for RNA splicing.

To further verify this conclusion, we examined three more E6E7 pre-mRNAs having a smaller, but variable sizes of exon 2 with or without a U1 binding site attached to the RNA 3′ end, for *in vitro* splicing (Fig. S2E). We demonstrated that all tested pre-mRNAs with a small exon 2 plus an U1 binding site were spliced efficiently at the nt 409 3′ss (Fig. S2F, compare pre-mRNAs 4, 6, and 8 to pre-mRNAs 3, 5, and 7). Data indicate that a natural size of exon 2 in HPV16 E6E7 pre-mRNA restrains exon definition to promote RNA splicing.

### Mapping of the Branch Site(s) Controlling the nt 409 3′ ss Usage in HPV16 Intron 1 Splicing

**Figure 4:**
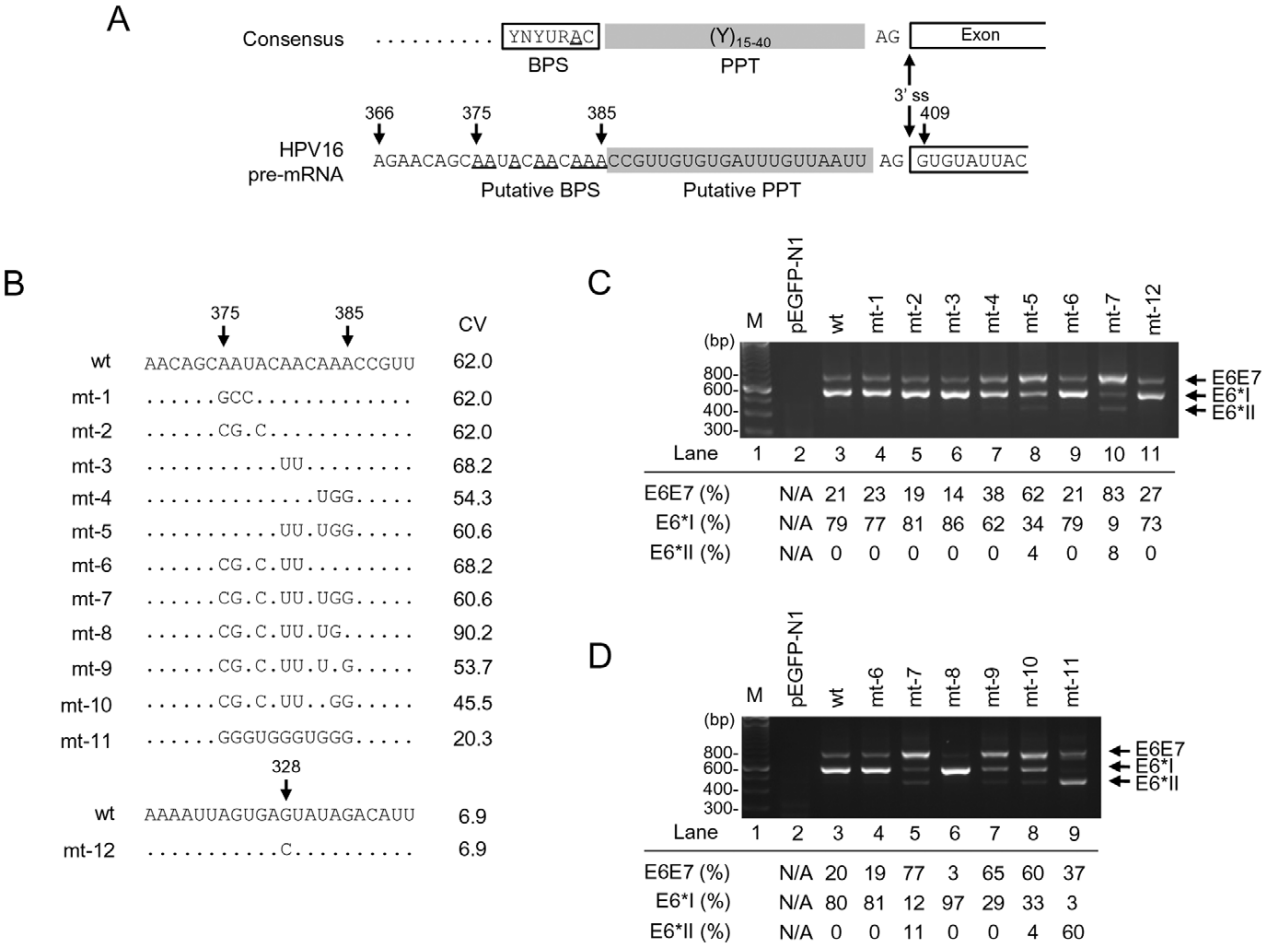
Mapping of the BPS in splicing of the HPV16 nt 409 3′ ss. (A) Consensus splicing elements (3′ ss, BPS, polypyrimidine tract [PPT]) in a typical 3′ ss (upper) and their corresponding RNA sequences in the HPV16 nt 409 3′ ss (lower). (B) RNA sequences of putative wild type (wt) BPS and its derived mutants (mt 1–11) in HPV16 E6E7 constructs used in the study. Arrow heads indicate the nucleotide position in the HPV16 genome. Unchanged ribonucleotides in each mutant are indicated by dots. A mutant G328C BPS reported by another lab [46] was included in this study for verification. The nucleotide G at nt 328 position in the HPV16 genome was substituted with a C in the mutant construct (mt-12). CV, consensus value of the putative BPS [23]. (C and D) Identification of the nt 385A as a preferential branch site for splicing of the nt 409 3′ ss. RT-PCR results were obtained by using a primer pair of Pr106 and Pr855 (Fig. 2A) from total RNA extracted from HEK293 cells 24 h after transfection with a wt or mt construct as indicated above the agarose gel. Vector pEGFP-N1 served as a negative control (lane 2 in both C and D). M, size marker of 100-bp DNA ladders (lane 1 in both C and D). Identities of PCR products derived from unspliced E6E7 and spliced E6*I and E6*II RNA are shown on the right. Relative ratios (%) of the unspliced E6E7 (intron 1 retention) and spliced E6*I (nt 226∧409) and E6*II (nt 226∧526) RNA were calculated by the signal intensity of each product and shown below the gel.

We next focused on mapping of the other essential cis-elements, including a BPS and PPT, in the HPV16 intron 1 that regulate the recognition of 3′ ss during RNA splicing. As we described above, an intron 5′ ss is recognized by U1 snRNP, while recognition of a 3′ ss depends on U2 snRNP interaction with BPS through base paring and on U2AF65 association with a PPT immediately downstream of the BPS [35], [36]. In mammalians, PPT is usually in size of 12–40 nts immediately upstream of a 3′ ss [37]–[39] and an adenosine residue in BPS at an appropriate distance from the 3′ ss is the primary determinant of the lariat-intermediate formation for the second catalytic step of RNA splicing [40]. Thus, elucidation of a BPS position containing a critical adenosine to connect with the intron 5′ end guanosine through the 5′-to-2′ phosphodiester bond in the lariat formation would define a particular PPT size. By scanning the sequence immediately upstream of the nt 409 3′ ss, we identified a 21-nt pyrimidine-rich stretch which is interspersed with four guanosines and three adenosines, followed by an A run of the sequence further upstream (Fig. 4A). We hypothesized that this A-run sequence from nt 375 to 385 could be a putative BPS region for splicing of the nt 409 3′ ss (Fig. 4A). This putative BPS appears to be deviated from the mammalian consensus BPS, YNYURAC, in which the 6th adenosine (underlined) could be bulged in the first catalytic step [41]–[43]. However, when compared with the mammalian consensus BPS, YNYURAC, the putative BPS region shows only a suboptimal match to the consensus, with the nt 385 A as a putative branch site having the best consensus value (CV) of 62 (Table S4). To pinpoint the branch site for splicing of the HPV16 intron 1, we initially applied Superscript II RT-PCR [19], [44] and primer extension [45] techniques, two well-established branch point mapping methods, but failed. We then introduced a series of point mutations into the putative BPS region of HPV16 E6E7 expression vector (Fig. 4B). A G-to-C mutation at nt 328 position (Fig. 4B) was also constructed because the G nucleotide at the nt 328 was reported previously by another laboratory to be a putative branch site for splicing of the nt 409 3′ss [46]. After transfection of HEK293 cells, HPV16 E6E7 RNA splicing from each expression vector was profiled by RT-PCR using a primer pair of Pr106 and Pr855 as indicated in Fig. 2A. As expected, splicing from nt 226 to nt 409 to produce E6*I mRNA was predominant over the splicing from nt 226 to nt 526 (Figure 4C and 4D, lanes 3) in the 293 cells transfected by an HPV16 E6E7 expression vector containing the putative wt BPS region. Similarly, introduction of point mutations in the putative BPS in the expression vectors mt-1, mt-2, mt-3, and mt-6 did not change the splicing profile (Fig. 4C, lanes 4–6 and lane 9), indicating that the adenosines from nt 375 to nt 381 in the putative BPS region do not function as a branch site. We also found that a G-to-C mutation at the nt 328 position showed no effect on E6*I production (Fig. 4C, lane 11). In fact, the guanosine at nt 328 lacks a juxtaposed downstream PPT and has four additional AG dinucleotides upstream of the nt 409 3′ss. Thus, the proposed nt 328G as a branch site [46] does not fit into the splicing scanning model by recognizing the first AG dinucleotide 3′ of the branch site [35], [47]–[49]. Our result in Fig. 4C (lane 11) indicates that the nt 328G reported in other study [46] is not an authentic branch site. Interestingly, we observed a reduced E6*I splicing in the HEK293 cells transfected with the expression vector mt-4 containing adenosine mutations from the nt 383 to nt 385, where E6*I production level reduced to 62% (Fig. 4C, lane 7) from wt 79% (Fig. 4C, lane 3) accompanied by a slight increase (from wt 21% to 38%) of unspliced E6E7, indicating that the As from the nt 383 to 385 appear to be important for splicing of the nt 409 3′ ss. This became more obvious in the mt-5 where these A mutations were combined with two additional A mutations at both nt 380 and nt 381, although the latter two mutations alone in an expression vector mt-3 did not affect the splicing. We found a more than 50% reduction of E6*I production and a ∼3-fold increase of unspliced E6E7 from the mt-5-transfected cells (Fig. 4C, compare lane 8 to lane 3). Data suggest the presence of alternative branch site selection in the putative BPS, where the AAA from nt 383 to 385 could be a primary choice in E6*I splicing. However, when these three adenosines were mutated, the upstream adenosines at the nt 380 and nt 381 could be activated as a branch site for E6*I splicing. Moreover, the cells transfected with an expression vector mt-7 which contains all adenosine mutations from nt 375 to 385, showed almost no production (only 9%) of E6*I, but 83% of unspliced E6E7 RNA along with an increased production (8%) of E6*II (Fig. 4C, lane 10). These data further indicate that the adenosines from nt 375 to nt 378 could be alternatively utilized as a branch site for E6*I splicing when the adenosines from nt 380 to 385 are blocked.

Given the fact that the expression vector mt-7 was only minimally spliced (Fig. 4C, lane 10) and the mt-6 differing from the mt-7 by remaining three As unchanged from the nt 383 to 385 was highly spliced (Fig. 4C, lane 9) in transfected HEK293 cells, we next examined which adenosine from nt 383 to 385 is preferentially selected as a branch site for splicing of the nt 409 3′ ss. For this purpose, the adenosine from nt 383 to 385 were individually restored from the mt-7, leading to construction of three additional expression vectors mt-8, mt-9 and mt-10 and each differing from the mt-7 only by single nucleotide at nt 385 (mt-8), 384 (mt-9), or 383 (mt-10) (Fig. 4B). When these constructs were compared with the mt-7, mt-6, and wt expression vectors in transfected HEK 293 cells for splicing of HPV16 intron 1, we found that restoration of the nt 385A in mt-8 led to drastic E6*I splicing as seen from the mt-6 and wt vector (Fig. 4D, compare lane 6 to lanes 5, 4, and 3). On the other hand, restoration of the nt 384A (mt-9) or nt 383A (mt-10) resulted in only a moderate increase in E6*I splicing (Fig. 4D, compare lanes 7–8 to lane 5). Together, these data indicate that the nt 385A which is most proximal to the nt 409 3′ ss is the primary branch site. We also examined an expression vector mt-11 with the lowest CV score in the correspondent putative BPS region (Fig. 4B) for E6*I splicing. We demonstrated that this construct expressed very little E6*I, but a remarkable amount of E6*II (Fig. 4D, lane 9), further indicating that this expression vector with a disrupted BPS has an inactive nt 409 3′ ss leading to a downstream nt 526 3′ ss being highly active.

### Introduction of Point Mutations into the Mapped BPS in the nt 409 3′ss Inhibits in vitro RNA Splicing of HPV16 E6E7 Pre-mRNA

**Figure 5:**
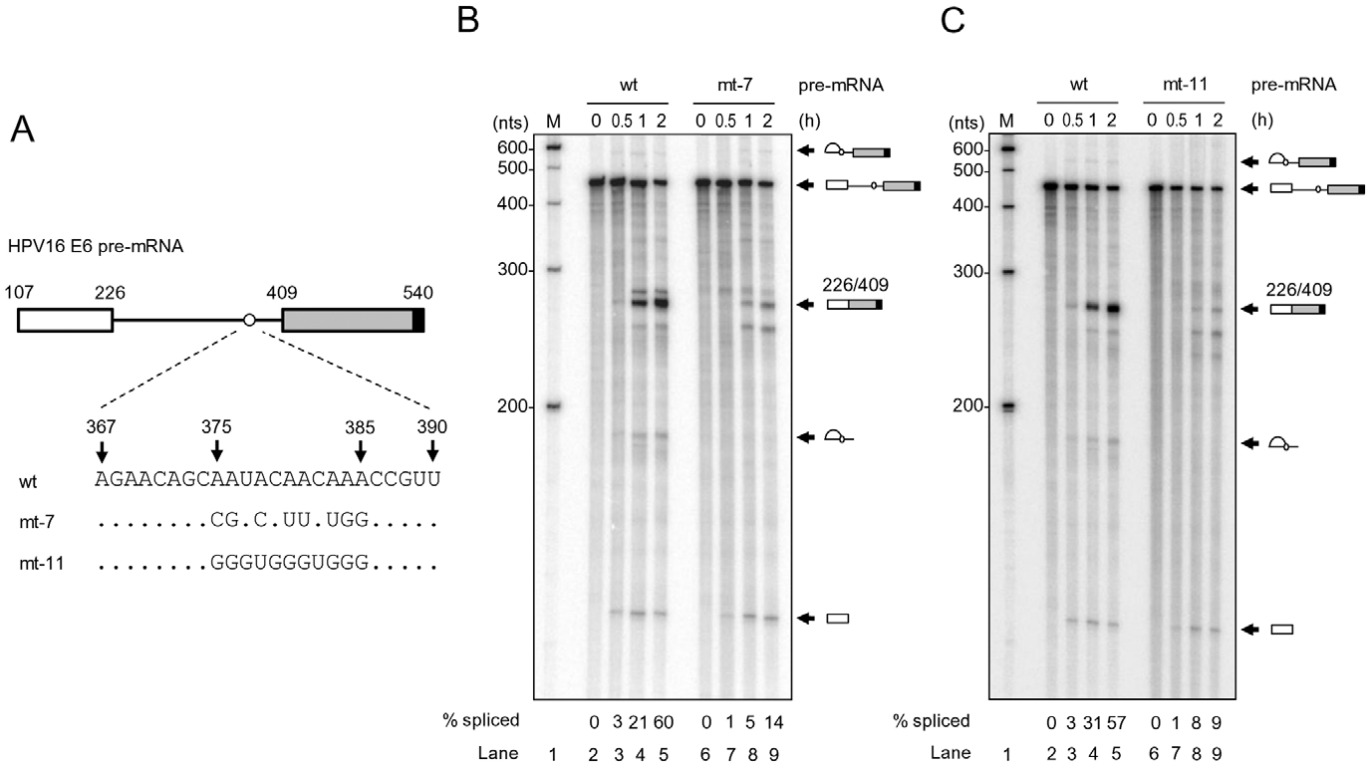
Introduction of point mutations into the mapped BPS in HPV16 intron 1 inhibits RNA splicing *in vitro*. (A) Diagram of HPV16 pre-mRNA used in *in vitro* splicing assays. First exon (nt 107–226) and second exon (nt 409–540), indicated by white and gray rectangles, respectively, are separated by its native intron 1 containing a wt, mt-7 or mt-11 BPS. An 11-nt U1 binding site (black rectangle) attached to each RNA 3′ end served as a 5′ ss or splicing enhancer to promote *in vitro* RNA splicing. (B and C) Reduction of HPV16 RNA splicing *in vitro* by introduction of point mutations into the mapped BPS. *In vitro* RNA splicing assay was performed with ^32^P-labeled HPV16 E6 pre-mRNA in the presence of HeLa nuclear extract at 30°C for the indicated splicing reaction time (h). Spliced products were resolved in a 6% denaturing PAGE gel and their identities are shown on the right. Splicing efficiency (% spliced) was calculated as described [24] from each spliced gel and is shown at the bottom of the gel.

To further analyze the effect of BPS mutation on HPV16 intron 1 splicing, we performed *in vitro* splicing assays. ^32^P-labeled HPV16 E6E7 pre-mRNAs harboring a wt, mt-7 or mt-11 BPS were synthesized by *in vitro* transcription (Fig. 5A). The pre-mRNAs used in the assays had a smaller exon 2 and were attached with a consensus 5′ ss at the RNA 3′-end (see Fig. S1) to promote the recognition of nt 409 3′ ss [8]. As shown in Fig. 5B and 5C, the pre-mRNA with a wt BPS was efficiently spliced (60% in Fig. 5B and 57% in Fig. 5C) by 2 h in our splicing conditions and produced a fully spliced product in size of ∼263 nts (see lanes 2–5 in Fig. 5B and 5C). Consistent with our observations described in Fig. 4B–4D, mt-7 pre-mRNA with all adenosine mutations in the mapped BPS was spliced poorly in vitro and the majority (86%) of the mt-7 pre-mRNA remained unspliced by 2 h under our splicing conditions (Fig. 5B, lanes 6–9). Similarly, mt-11 pre-mRNA with mutations in the mapped BPS also showed very little RNA splicing, with 91% of the pre-mRNAs left unspliced by 2 h (Fig. 5C, lanes 6–9). Unexpectedly, we consistently observed a similar amount of the cleaved exon 1 in size of ∼120 nts from the first step of RNA splicing among all three tested pre-mRNAs (wt, mt-7 and mt-11, Fig. 5B and 5C), implying that disruption of the mapped BPS affects mainly the second-step RNA splicing of HPV16 E6E7 intron 1 as described for rabbit β-globin pre-mRNA [50] and a yeast intron [51].

### Determination of U2 Interaction with the Mapped BPS in the HPV16 nt 409 3′ ss by RNase H Protection Assays

**Figure 6:**
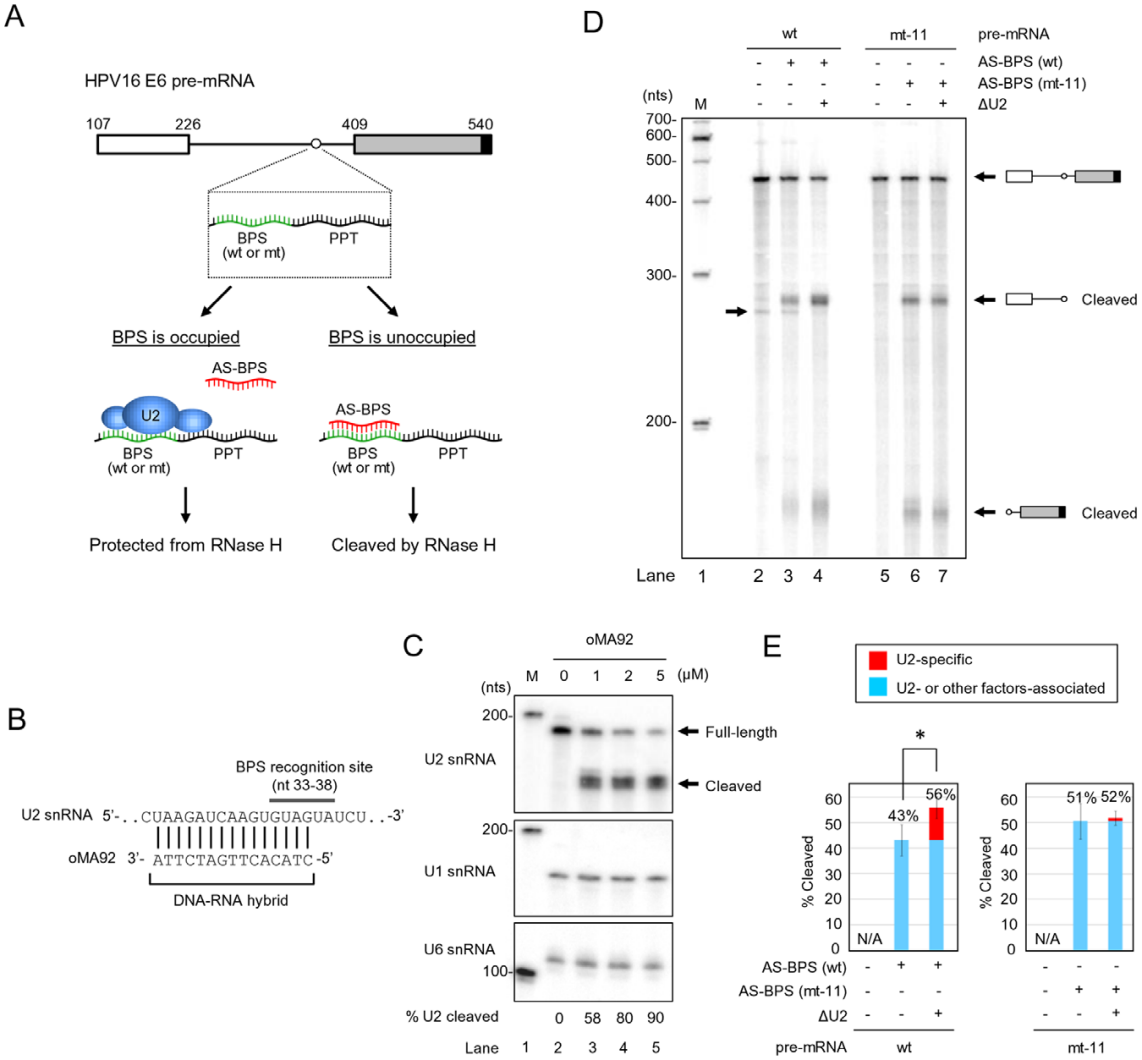
RNase H digestion to quantify U2 snRNP association with the wt or mt-11 BPS in the HPV16 intron 1. (A) Flow chart of RNase H protection assays. A ^32^P-labeled HPV16 E6 RNA with a wt or mt-11 BPS was incubated with HeLa nuclear extract with or without U2 depletion in the presence of an antisense (AS) DNA oligo to wt or mt-11 BPS and RNase H. Association of U2 snRNA or other factors (unlabeled ovals) with the mapped BPS prevents the antisense DNA oligo to specifically access the correspondent region, thereby preventing RNase H cleavage of the testing pre-mRNA. Otherwise, binding of the antisense DNA oligo to the mapped BPS would form a DNA-RNA duplex and trigger RNase H digestion. (B) Diagram of DNA oligo-mediated U2 depletion from HeLa nuclear extract by RNase H digestion. A DNA oligo (oMA92) antisense to U2′s BPS recognition site was incubated at 30°C with HeLa nuclear extract for 30 min and the mixture was then digested by RNase H at 30°C for additional 10 min. (C) Determination of U2 depletion efficiency from HeLa nuclear extract by Northern blot. Total RNA from HeLa nuclear extract with or without U2 depletion by U2 DNA oligo-mediated RNase H digestion was quantified by Northern blot with a ^32^P-labeled DNA oligo specific for U2 (oKY50). U1 and U6 served as loading controls. U2 depletion efficiency was expressed as a ratio (%) of cleaved U2 vs remaining full-length U2 dividing by total signal intensity of U2 in each reaction condition of RNase H digestion. (D and E) U2 interaction with the mapped BPS prevents oligo-directed RNase H digestion. HeLa nuclear extracts with or without U2 depletion (ΔU2) were compared for DNA-oligo-mediated RNase H cleavage of HPV16 pre-mRNA with a wt or mt BPS (A). The reaction products were resolved in a 6% denaturing PAGE gel. Identities of RNase H cleavage products are shown on the right of the gel (D). An arrow on the left of the gel (D) indicates a spliced product from the wt pre-mRNA during 30 min incubation of RNase H digestion in the presence of HeLa nuclear extract. Cleavage efficiency (%) (E) of HPV16 E6 pre-mRNA with a wt or mt BPS from DNA oligo-mediated RNase H digestion in each reaction was calculated from four independent experiments by the sum of signal intensity of all cleavage products divided by the sum of signal intensity of both full-length pre-mRNA and cleavage products. *, P<0.05 by Student′s t-test.

Association of U2 snRNP with a BPS has been thought to be one of the essential steps for bridging 5′ ss and bulged adenine residue in the BPS via a 5′-to-2′ phosphodiester bond. To determine the interaction of U2 snRNP with the mapped BPS in the HPV16 E6E7 intron 1, we then examined this interaction by an RNase H protection assay. This method is based on the catalytic activity of RNase H to specifically digest the testing RNA at the 3′ end of a DNA-RNA duplex region when an antisene DNA oligo base-pairs with a naked RNA region [26]. To this end, a ^32^P-labeled HPV16 E6 RNA with either a wt or mt BPS (mt-11) in the intron 1 was incubated with HeLa nuclear extract with or without U2 depletion at 30°C for 30 min in the presence of an antisense DNA oligo to wt or mt BPS. The mixtures were then subject to RNase H digestion (Fig. 6A). U2 depletion from HeLa nuclear extract was carried out also by RNase H digestion in the presence of an antisense DNA oligo to U2′s BPS recognition site (Fig. 6B) and was efficient (up to 90%) in our experimental condition (Fig. 6C). As U2 and other BPS-binding proteins in nuclear extract compete with the antisense DNA oligo for the binding site in the mapped BPS and therefore binding of these cellular factors to the mapped BPS would prevent DNA oligo-directed RNase H digestion. In contrast, if it is free from binding of cellular factors, the mapped BPS could be accessed by its corresponding antisense DNA oligo and thereby digested by RNase H. Subsequently, efficient U2 association with the mapped wt BPS or its mutant was evaluated by comparing RNA cleavage efficiency of the oligo DNA-directed RNase H digestion. As shown in Fig. 6D and 6E, we demonstrated that HPV16 E6E7 pre-mRNA in the presence of an antisense DNA oligo to the mapped BPS exhibited less digestion than its correspondent RNA with a mt-11 BPS in the presence of an antisense DNA oligo to the mt BPS (compare lane 3 to lane 6 in Fig. 6D and their corresponding columns in Fig. 6E). This level of digestion efficiency of wt BPS was greatly increased in the presence of U2-depleted HeLa nuclear extract (compare lane 3 to lane 4 in Fig. 6D and their corresponding columns in Fig. 6E). In contrast, the pre-mRNA containing mt-11 BPS displayed not only higher digestion efficiency in the same RNase H digestion condition, but appeared no correlation to U2-depletion (Fig. 6E). These data clearly indicate that the mapped BPS favors the binding of U2.

A pre-mRNA containing mt-7 BPS was also tested separately (Fig. S3) and showed ∼16% increase of RNase H digestion over the pre-mRNA containing wt BPS (Fig. S3B, compare lane 5 to lane 4), further confirming the mapped BPS as a preferential bind site for U2. On the other hand, we noticed that the PPT of both wt and mt-7 RNA was completely protected from RNase H cleavage in the presence of an antisense DNA oligo to the respective region (Fig. S3B, lanes 6–7) and the wt BPS pre-mRNA spliced during the 30 min incubation of RNase H digestion (Fig. S3, lanes 2, 4 and 6). Data suggest that introduction of point mutations into the mapped BPS affects the binding of U2 to the BPS, but not U2AF65 [52]–[54] to the PPT of the nt 409 3′ ss.

### Biological Significance of the Mapped BPS in the Expression of E6 and E7 Oncogenes

**Figure 7:**
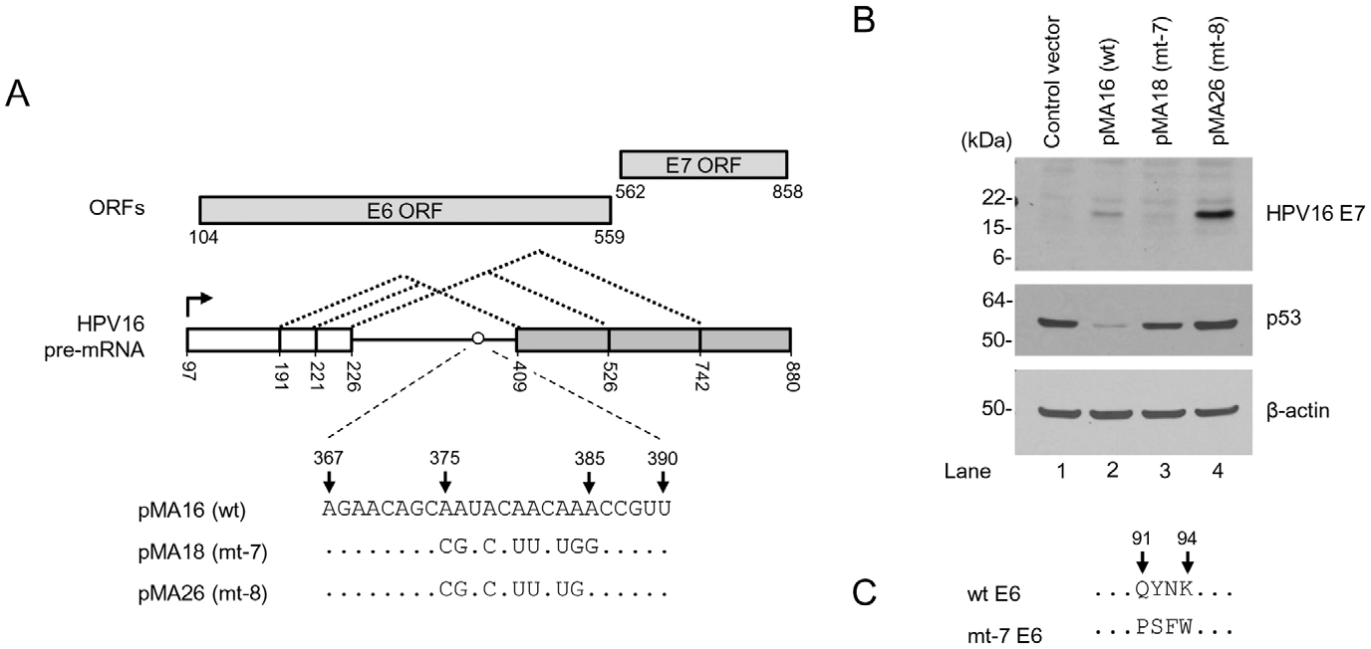
Biological significance of the mapped BPS in the expression of E6 and E7 oncogenes. (A) Diagrams of HPV16 E6E7 pre-mRNA expression vectors. Plasmid pMA16 has wild type sequence of E6E7 pre-mRNA, pMA18 expresses mt-7 pre-mRNA with a mt BPS, and pMA26 expresses mt-8 pre-mRNA similar to mt-7, except the 385A being restored as in wt. See RNA splicing profiles from each vector in Fig. 4D. (B) The expression of viral E6 and E7 oncoprotein from plasmid pMA16 (wt), pMA18 (mt-7), and pMA26 (mt-8). HCT116 cells were analyzed by Western blot 48 h after transfection with the indicated vectors above the gel for viral E7 and p53 (as an indicator of viral E6 expression and its activities). β-actin served as a loading control. (C) Amino acid residues in mt-7 E6 differs from wt E6 from aa 91 to 94 positions. Dots, unchanged aa residues.

As we recently reported, HPV16 E6 and E7 expression is regulated by RNA splicing and viral E7 could be only expressed efficiently from the spliced E6*I RNA [13]. Given the fact that the intorn 1 BPS identified in this study is crucial for E6*I splicing, we examined how the altered intron 1 splicing by the BPS mutation affects the expression of the viral oncogenes. We transfected HCT116 cells harboring wt p53 and wt pRb [55], [56] with an E6E7 expression vector pMA16 (wt), pMA18 (mt-7) or pMA26 (mt-8) as diagramed in Fig. 7A. An empty vector was used as a negative control for the transfection. The levels of viral oncogene expression were examined 48 h after the transfection by Western blot. HPV16 E6 expression was measured by p53, a downstream target of viral E6 [57], because of the lack of a suitable antibody for E6 detection. As shown in Fig. 7B, we found that expression of E6 and E7 oncogenes was closely correlated with viral RNA splicing as reported [13]. The pre-mRNA transcribed from the wt expression vector pMA16 spliced at a ratio of 1∶4 (unspliced E6E7:spliced E6*I) (Fig. 4C and 4D, lanes 3) and expressed both E6, as indicated by reduction of p53 level (Fig. 7B, compare lane 2 to lane 1), and E7 (Fig. 7B, lane 2). The mt-7 transcript containing a mt BPS derived from the pMA18 displayed inefficient RNA splicing with a ratio of unspliced E6E7 vs spliced E6*I at 6–9∶1 (Fig. 4C, lane 10 and Fig. 4D, lane 5) and expressed very little E7 (Fig. 7B, lane 3). This was expected because only the E6*I RNA supports an efficient translation-reinitiation of a scanning ribosome for E7 translation [13]. Interestingly, we found that viral E6 translated from the unspliced mt-7 RNA has four amino acid (aa) substitutions from aa 91 to 94 of wt E6 (Fig. 7C) and this mt E6, when compared with wt E6, appeared less activity to degrade p53 in the cells (Fig. 7B, compare lane 3 to lane 1). The mt-8 pre-mRNA transcribed from the pMA26 having the nt 385A restoration on the background of mt-7 spliced efficiently at a ratio of 1∶32 (unspliced E6E7:spliced E6*I) (Fig. 4D, lane 6) and expressed high level of E7 (Fig. 7B, lane 4). Collectively, our findings clearly indicate the biochemical and biological importance of the mapped BPS in splicing of HPV16 intron 1 for the expression of viral E6 and E7 oncogenes.

## Discussion

**Figure 8:**
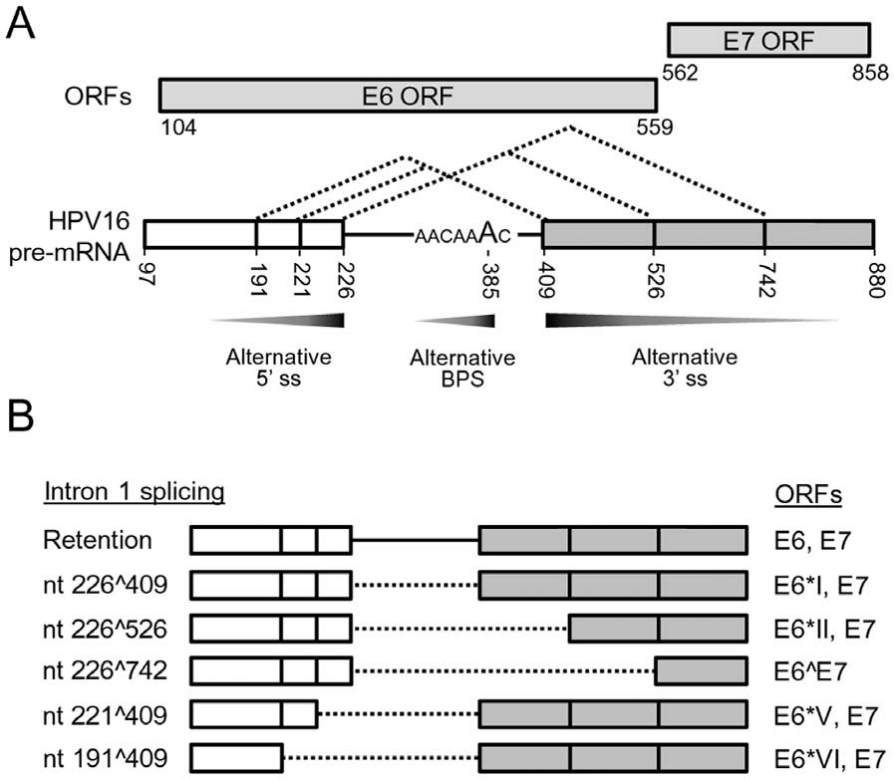
Summary of the characterized features of HPV16 E6E7 intron 1. (A) Preferential selection of a proximal splice site and branch site in splicing of the HPV16 E6E7 intron 1 which contains three suboptimal 5′ ss (nt 191, 221 and 226), three suboptimal 3′ ss (nt 409, 526 and 742), and a cluster of branch site As from nt 383 to nt 385. In this regard, a proximal nt 226 5′ ss is preferentially selected over the nt 221 and nt 191 5′ ss, a proximal nt 409 3′ ss is efficiently selected over the nt 526 and nt 742 3′ ss, and the branch site A at nt 385 is preferentially utilized over the nt 384 A or 383 A, to minimize splicing energy and length of the excising intron 1. The preferential selection of an alternative 5′ ss, 3′ ss and branch site is exercised by the principle of proximity and is indicated by graded grey arrows for more (dark grey) to less (light grey) splicing of the HPV16 E6E7 intron 1. (B) Alternative splicing of HPV16 E6 intron 1 leads to produce multiple E6 RNA species detectable in cervical cancer tissues and their derived cell lines. Dash lines indicate the spliced intron 1 from each isoform of E6E7 RNA and vertical lines in white (exon 1) and grey boxes (exon 2) stand for splice sites. ORFs for splicing variants of viral E6 and E7 are indicated on the right.

Viruses have evolutionarily developed a most cost-effective way to maximize their utilization of host machinery for their genome replication and gene expression. Papillomaviruses have adapted their life cycle tightly to keratinocyte differentiation by differential usage of alternative promoters, splice sites, and poly (A) signals for viral early and late gene expression [7], [58], [59]. One of the most complicated features of papillomavirus gene expression is alternative RNA splicing of viral bicistronic or polycistronic RNAs. In BPV-1 late gene expression, viral RNA cis-elements are involved in selection of alternative splice sites by interaction with cellular splicing factors differentially expressed in keratinocytes [7]. Similar to BPV-1, HPV16 also expresses its early and late genes through the alternative RNA splicing which is also governed by viral RNA cis-elements and cellular splicing factors [59]. In this report, we have systematically characterized HPV16 intron 1 and its cis-elements governing alternative splicing of viral E6E7 bicistronic RNA. We identified two novel 5′ ss (nt 221 5′ ss and nt 191 5′ss) and their normal usage in cervical cancer and its derived cell lines, thereby revealing the presence of three alternative 5′ ss in addition to three known alternative 3′ ss in the HPV16 intron 1. All of these splice sites identified in the HPV16 intron 1 are suboptimal. Our finding that the adenosine at nt 385 position in the HPV16 genome serves as a major branch site for selection of the nt 409 3′ ss for E6*I splicing and viral E7 expression paves a way for the first time for us to understand RNA splicing in regulation of viral E6 and E7 expression from the intron 1 region. Most importantly, we provide the evidence that selection among three alternative 5′ ss and three alternative 3′ ss of HPV16 E6E7 intron 1 is achieved by a simple principle of the proximity where a minimal length of the intron is preferentially excised (Fig. 8).

Proximal pairing of donor and acceptor sites is known as the proximal rule in RNA alternative splicing and was initially reported in 1986 [60]. Although multiple factors, including cis-elements, distance between splice sites and length of intron, might be important for the proximal rule, it remains unknown what contributes predominantly to the principle and how each splice site could be distinguished during the preferential selection. In case of the HPV16 intron 1, three alternative 5′ ss are closely located each other, but the nt 226 5′ ss is preferentially selected over the other two, presumably due to its relatively higher base-pairing affinity to U1 as demonstrated by ESEfinder analysis (http://rulai.cshl.edu/ESE). U1 pairing efficiency was reported as an critical factor for selection of the proximal 5′ ss when a competing 5′ ss is separated by <80 nucleotides [61], [62]. On the other hand, selection of an alternative 3′ ss could be more complex and it involves intronic and/or exonic cis-elements, strength and proximity of PPT and BPS, and strength of the intron 3′ end (YAG) [39], [49], [63]. Although showing less strength than the nt 526 3′ ss by the ESEfinder, the HPV16 nt 409 3′ ss is preferentially selected for viral E6E7 splicing, most likely due to the presence of a functional BPS positioned 20 nts upstream of the 3′ AG dinucleotide. Supporting this notion, we showed that the second step splicing of viral E6E7 intron 1, but not its first step of splicing, could be affected by A-to-G mutation in the nt 385 position (Fig. 5) as described for other pre-mRNAs [50], [51]. Thus, our data is consistent with the observation that a BPS-proximal 3′ ss is preferentially selected as the result of 5′ to 3′ scanning process by recognizing the first AG dinucleotide downstream of the branch site [35], [47]–[49], [64].

Previous report suggested that the G at nt 328 in HPV16 E6E7 intron 1 might function as a putative branch site for splicing of the nt 409 3′ss [46]. However, we found that the HPV16 E6E7 expression vector with G-to-C mutation at the nt 328 position showed no effect on splicing of the nt 409 3′ ss, indicating that the nt 328G is not a functional BPS. Instead, we demonstrated that splicing of the nt 409 3′ ss is facilitated primarily by the adenosine at nt 385 position. The mapped BPS with a bulged A at the nt 385 for splicing of the nt 409 3′ ss is not consensus to YNYURAC as seen in many eukaryotic introns [65] although the mapped nt 385A has a better CV score than other adenosines immediately upstream (Table S4). When the nt 385A was mutated, only a small reduction of splicing efficiency of the nt 409 3′ ss was seen in our study. This result was somewhat expected because mutation of the nt 385A could activate the usage of the nt 384A, nt 383A, or even an adenosine from the nt 375 to 381 as an alternative branch site. Early study on β-globin BPS indicated that mutation of a normal branch site A to G or U could lead to an A residue one nucleotide upstream of the normal branch site being used instead [50]. The finding of the nt 385 A being a principle branch site over other adenosines immediately upstream is also consistent with a recent observation that the usage of alternative branch sites has strong preference to the most downstream branch site adenosine [65].

The BPS is required for pairing with U2 and is important for the second step of RNA splicing and choice of the first AG 3′ ss downstream of the branch site [35]. Thus, the mapped BPS was examined in our report for its association with U2 snRNP in HeLa nuclear extract by an RNase H protection assay in the presence of a BPS-specific antisense DNA oligo. We demonstrated a specific association of U2 with the mapped BPS, leading to prevent DNA oligo-directed RNase H digestion. This was evidenced by the observation of an increased RNase H digestion in the E6E7 pre-mRNA bearing a mutant BPS (Fig. 6 and Fig. S3). The latter was further verified by using an U2-deficient HeLa nuclear extract (Fig. 6D and 6E). However, we found that other factors interact with the mapped BPS region and could prevent the E6E7 pre-mRNA from RNase H digestion (Fig. 6 and Fig. S3). As a result, depletion of U2 from HeLa nuclear extract enhanced the digestion efficiency of the wt BPS pre-mRNA only by 13% (Fig. 6E) over the nuclear extract without U2 depletion. This was expected since the BPS region is also the binding site for several cellular splicing factors. Base-pairing of U2 with the BPS is a highly ordered process involving several sequential steps and active participation of those splicing factors. It has been proposed that splicing factor 1 (SF1) initially binds the BPS prior to U2 and interacts with U2AF65 bound to an adjacent PPT to increase its binding affinity [66]–[70]. Subsequently, U2 base-pairing with the BPS displaces SF1 from the BPS [71], [72] and follows by binding of U2AF65-interacting, multisubunit SF3a and SF3b complexes to the sequences 5′ or 3′ of the BPS in a sequence-independent manner [73]–[76]. A recent study showed that SF1 also binds other RNA sequences not important for RNA splicing [69]. Nevertheless, identification of AACAAAC as the BPS for selection of the nt 409 3′ ss in this report clearly elucidates structural basis for the expression and regulation of HPV16 E6 and E7 oncogenes by alternative RNA splicing. More importantly, the alternative usage of a branch site and intron definition characterized for splicing of HPV16 intron 1 in this report appears to be a common feature from viruses and human.

By introduction of mutations into the mapped BPS region to prevent RNA splicing, we found that the unspliced E6E7 RNA from the mt-7 encodes a mutant E6 protein containing four aa substitutions (Q91P, Y92S, N93F and K94W). These aa substitutions in the mt-7 E6 was detrimental for E6 activity in degradation of p53 and presumably affect the structure and/or folding of HPV16 E6 protein [77], [78]. Although the F47 of HPV16 E6 was found to be the only amino acid residue required for E6 polyubiquitination and degradation of p53 [77], [79], our data clearly indicate that other regions of E6 protein in sustaining E6 folding and E6 structure might be also important for E6 activity.

## Supporting Information

Figure S1 **Sequencing results of RT-PCR products derived from HPV16 RNA spliced from nt 221 or 191 5′ ss to nt 409 3′ ss.** RT-PCR products from total RNA of the Ca 1 tissues or CaSki cells were amplified with a splice junction-specific primer pair of Pr221/409+ Pr562 for 221∧409 splicing and Pr191/409+ Pr562 for 191∧409 splicing (Figure 3A–C) and then gel-purified for sequencing by using primer Pr562. Sequence chromatograms of each splice junction region are shown, demonstrating the specific annualing of each splice junction primer to the splice junction of the spliced RNA. +dT, a thymine complimentary to the adenine added to 3′ end during PCR by Taq DNA polymerase.
(TIF)

Figure S2 **Exon definition promotes the splicing of HPV16 E6E7 pre-mRNA at nt 742 3**′ **ss** ***in vitro*** **.** (A) Structures of two HPV16 E6E7 pre-mRNAs used in *in vitro* splicing assay. Exons are indicated with white or gray boxes with a native 5′ ss (pre-mRNA 1) or 11 nt-consensus U1 binding site (black rectangle, pre-mRNA 2) at the RNA 3′ end. (B) Illustration of base-pairing of each RNA 3′ end (shaded, middle) with U1, U5 and U6 snRNAs, showing suboptimal base-pairing of the pre-mRNA 1 and optimal base-paring of the pre-mRNA 2. Ψ, pseudo-uridine; Gm3, 2,2,7-tri-methyl guanosine. (C) A splicing gel showing RNA splicing at nt 742 3′ss. *In vitro* splicing products were resolved in an 8% denaturing PAGE gel. The numbers above the splicing gel indicate the pre-mRNAs corresponding to panel A and splicing reaction time (h). Identities of the spliced products are shown on the right. (D) A band with the size corresponding to a splicing product from nt 226 5′ ss to nt 742 3′ ss in (C) was purified from the gel and sequenced, confirming the fully spliced product from nt 226 to 742. (E) Structures of other HPV16 E6E7 pre-mRNAs with a short exon 2 used in *in vitro* RNA splicing assay. See other descriptions in (A) for details. (F) A splicing gel showing RNA splicing at nt 409 3′ ss when the pre-mRNAs had a short exon attached by an U1 binding site.
(TIF)

Figure S3 **Disruption of the mapped BPS in HPV16 nt 409 3**′ **ss does not affect protein factors to interact with PPT.** (A) Strategy flow chart of RNase H protection assays to analyze U2 interaction with the mapped BPS and other cellular proteins interaction with PPT. A ^32^P-labeled HPV16 E6 RNA with a wt or mt-7 BPS was incubated with HeLa nuclear extract in the presence of an AS DNA oligo complimentary to wt or mt-7 BPS (AS-BPS), or downstream PPT (AS-PPT) for 10 min and followed by RNase H digestion. Association of U2 snRNP or other factors with BPS and U2AF65 with PPT prevent DNA oligo-mediated RNase H cleavage of the pre-mRNA. (B) RNase H digestion products were resolved in 6% denaturing PAGE gel containing 7.5 M Urea. Identities of cleavage products are indicated on the right side. An arrow on the left of the gel indicates a spliced 226∧409 product from 30 min incubation with HeLa nuclear reaction during RNase H digestion. Cleavage efficiency (%) was calculated as described in Fig. 6E.
(TIF)

Table S1 A list of expression vectors used in this study.
(TIF)

Table S2 A list of DNA oligomers used in this study.
(TIF)

Table S3 Prediction scores for 5′ ss and 3′ ss in HPV16 nt 97–880 by ESE finder.
(TIF)

Table S4 Branch point sequence consensus values in the nt 409 3′ ss of HPV16 intron 1.
(TIF)
