# Supplementary figures and images for: Intron Definition and a Branch Site Adenosine at nt 385 Control RNA Splicing of HPV16 E6*I and E7 Expression
Source: PLoS One. 2012 Oct 4;7(10):e46412. doi: 10.1371/journal.pone.0046412 (PMC3464268; doi:10.1371/journal.pone.0046412)

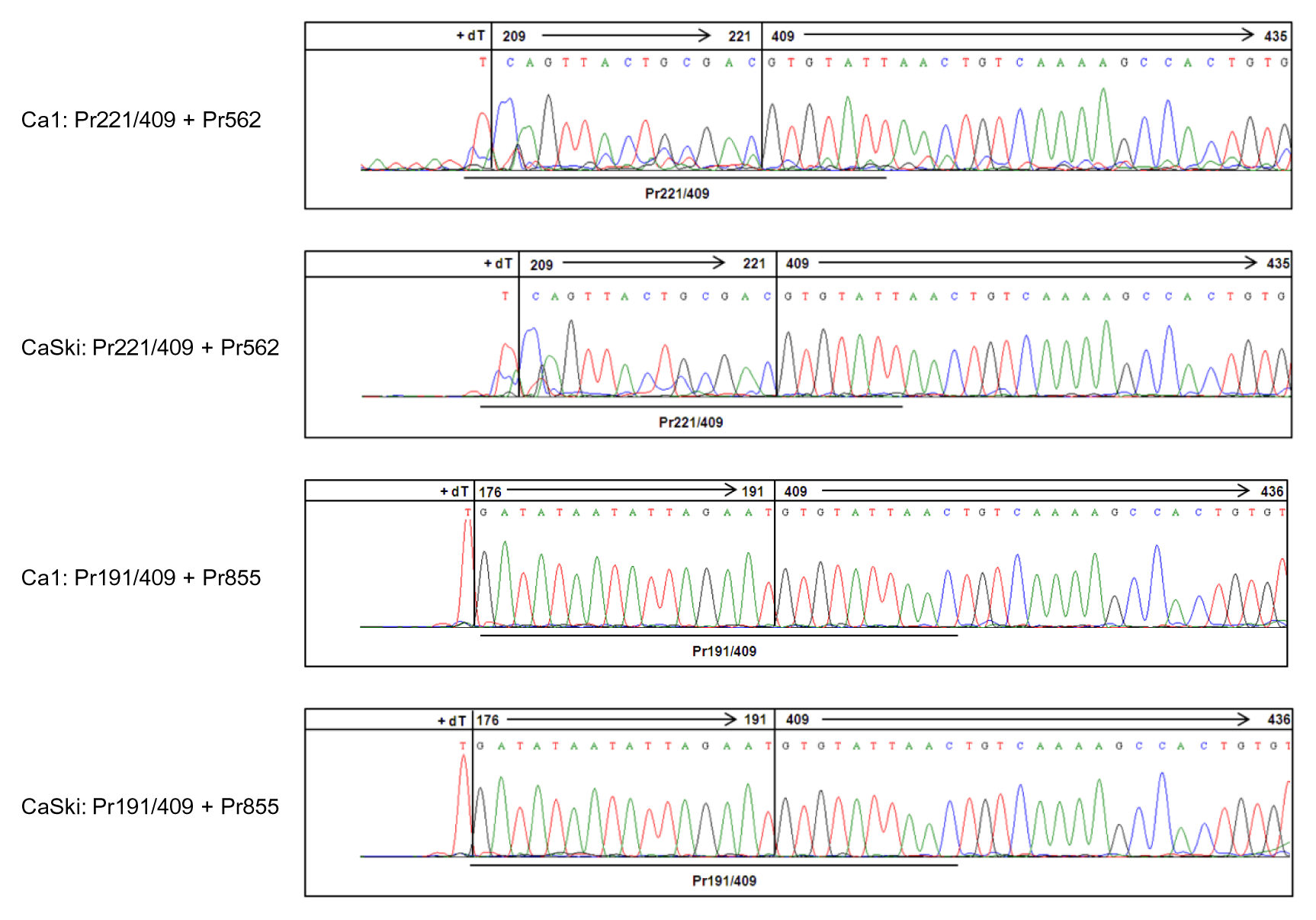

Supplement: Figure S1 — Sequencing results of RT-PCR products derived from HPV16 RNA spliced from nt 221 or 191 5′ ss to nt 409 3′ ss. RT-PCR products from total RNA of the Ca 1 tissues or CaSki cells were amplified with a splice junction-specific primer pair of Pr221/409+ Pr562 for 221∧409 splicing and Pr191/409+ Pr562 for 191∧409 splicing (Figure 3A–C) and then gel-purified for sequencing by using primer Pr562. Sequence chromatograms of each splice junction region are shown, demonstrating the specific annualing of each splice junction primer to the splice junction of the spliced RNA. +dT, a thymine complimentary to the adenine added to 3′ end during PCR by Taq DNA polymerase. (TIF) [file pone.0046412.s001.tif]

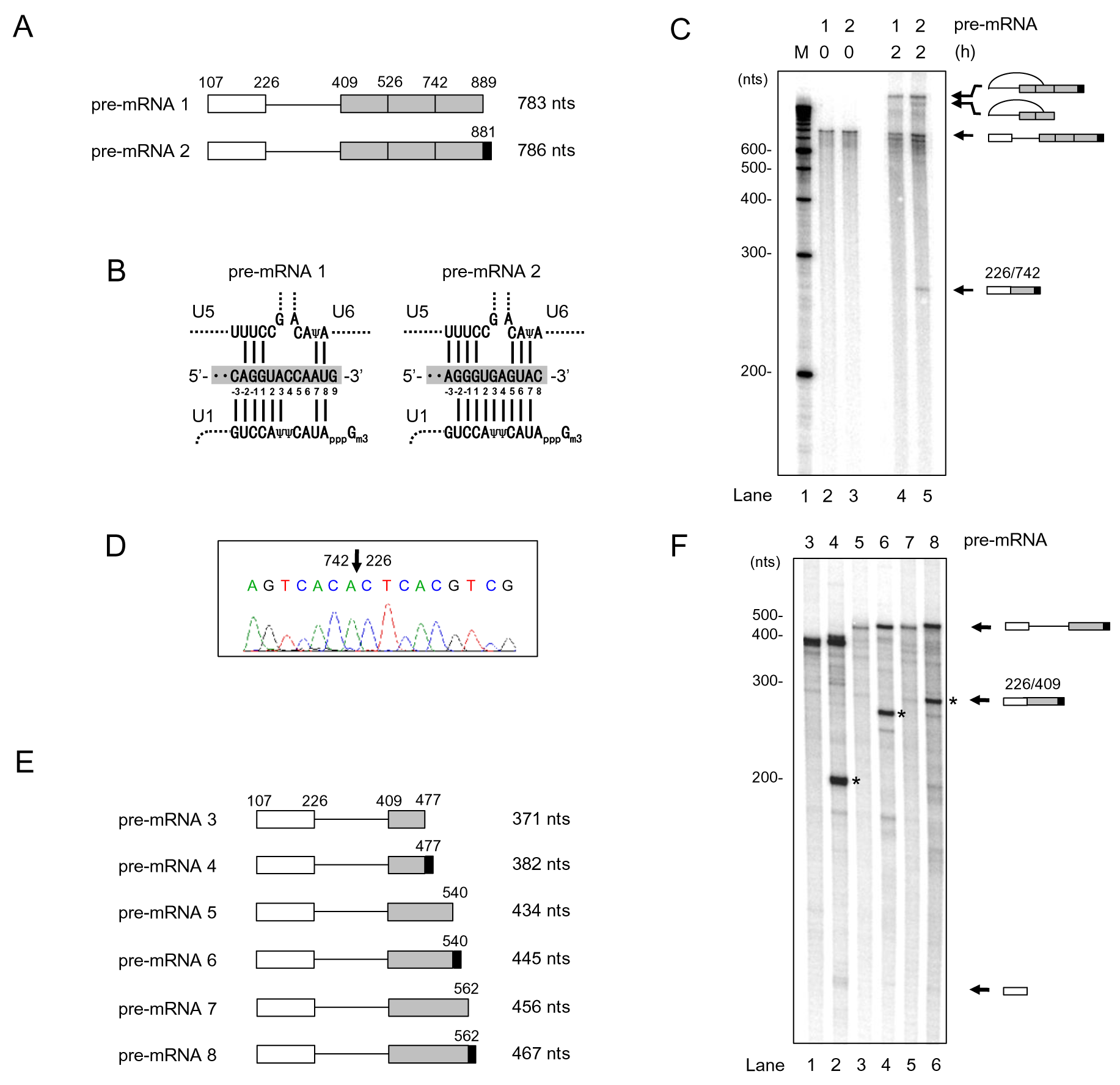

Supplement: Figure S2 — Exon definition promotes the splicing of HPV16 E6E7 pre-mRNA at nt 742 3′ ss in vitro . (A) Structures of two HPV16 E6E7 pre-mRNAs used in in vitro splicing assay. Exons are indicated with white or gray boxes with a native 5′ ss (pre-mRNA 1) or 11 nt-consensus U1 binding site (black rectangle, pre-mRNA 2) at the RNA 3′ end. (B) Illustration of base-pairing of each RNA 3′ end (shaded, middle) with U1, U5 and U6 snRNAs, showing suboptimal base-pairing of the pre-mRNA 1 and optimal base-paring of the pre-mRNA 2. Ψ, pseudo-uridine; Gm3, 2,2,7-tri-methyl guanosine. (C) A splicing gel showing RNA splicing at nt 742 3′ss. In vitro splicing products were resolved in an 8% denaturing PAGE gel. The numbers above the splicing gel indicate the pre-mRNAs corresponding to panel A and splicing reaction time (h). Identities of the spliced products are shown on the right. (D) A band with the size corresponding to a splicing product from nt 226 5′ ss to nt 742 3′ ss in (C) was purified from the gel and sequenced, confirming the fully spliced product from nt 226 to 742. (E) Structures of other HPV16 E6E7 pre-mRNAs with a short exon 2 used in in vitro RNA splicing assay. See other descriptions in (A) for details. (F) A splicing gel showing RNA splicing at nt 409 3′ ss when the pre-mRNAs had a short exon attached by an U1 binding site. (TIF) [file pone.0046412.s002.tif]

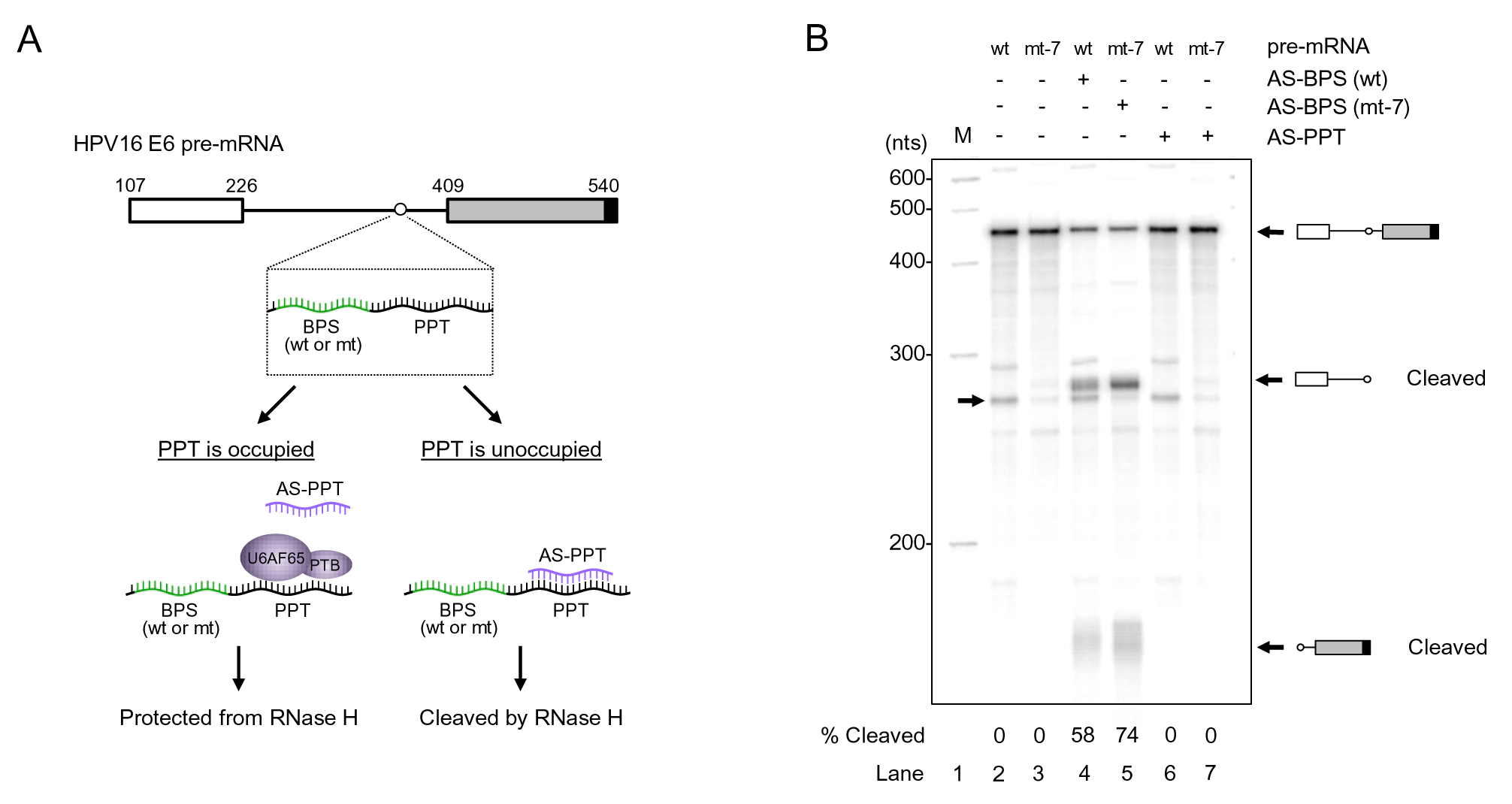

Supplement: Figure S3 — Disruption of the mapped BPS in HPV16 nt 409 3′ ss does not affect protein factors to interact with PPT. (A) Strategy flow chart of RNase H protection assays to analyze U2 interaction with the mapped BPS and other cellular proteins interaction with PPT. A 32P-labeled HPV16 E6 RNA with a wt or mt-7 BPS was incubated with HeLa nuclear extract in the presence of an AS DNA oligo complimentary to wt or mt-7 BPS (AS-BPS), or downstream PPT (AS-PPT) for 10 min and followed by RNase H digestion. Association of U2 snRNP or other factors with BPS and U2AF65 with PPT prevent DNA oligo-mediated RNase H cleavage of the pre-mRNA. (B) RNase H digestion products were resolved in 6% denaturing PAGE gel containing 7.5 M Urea. Identities of cleavage products are indicated on the right side. An arrow on the left of the gel indicates a spliced 226∧409 product from 30 min incubation with HeLa nuclear reaction during RNase H digestion. Cleavage efficiency (%) was calculated as described in Fig. 6E. (TIF) [file pone.0046412.s003.tif]

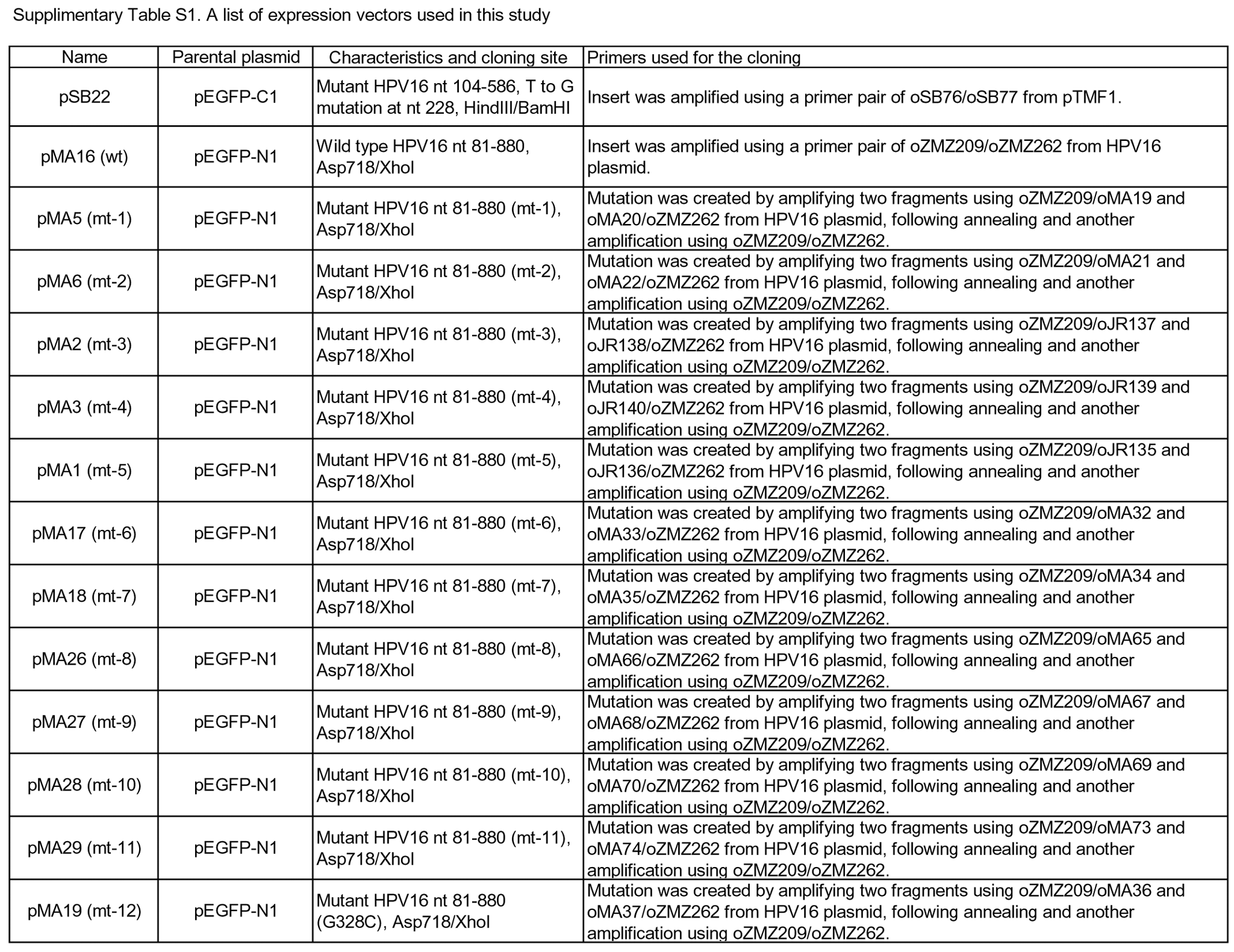

Supplement: Table S1 — A list of expression vectors used in this study. (TIF) [file pone.0046412.s004.tif]

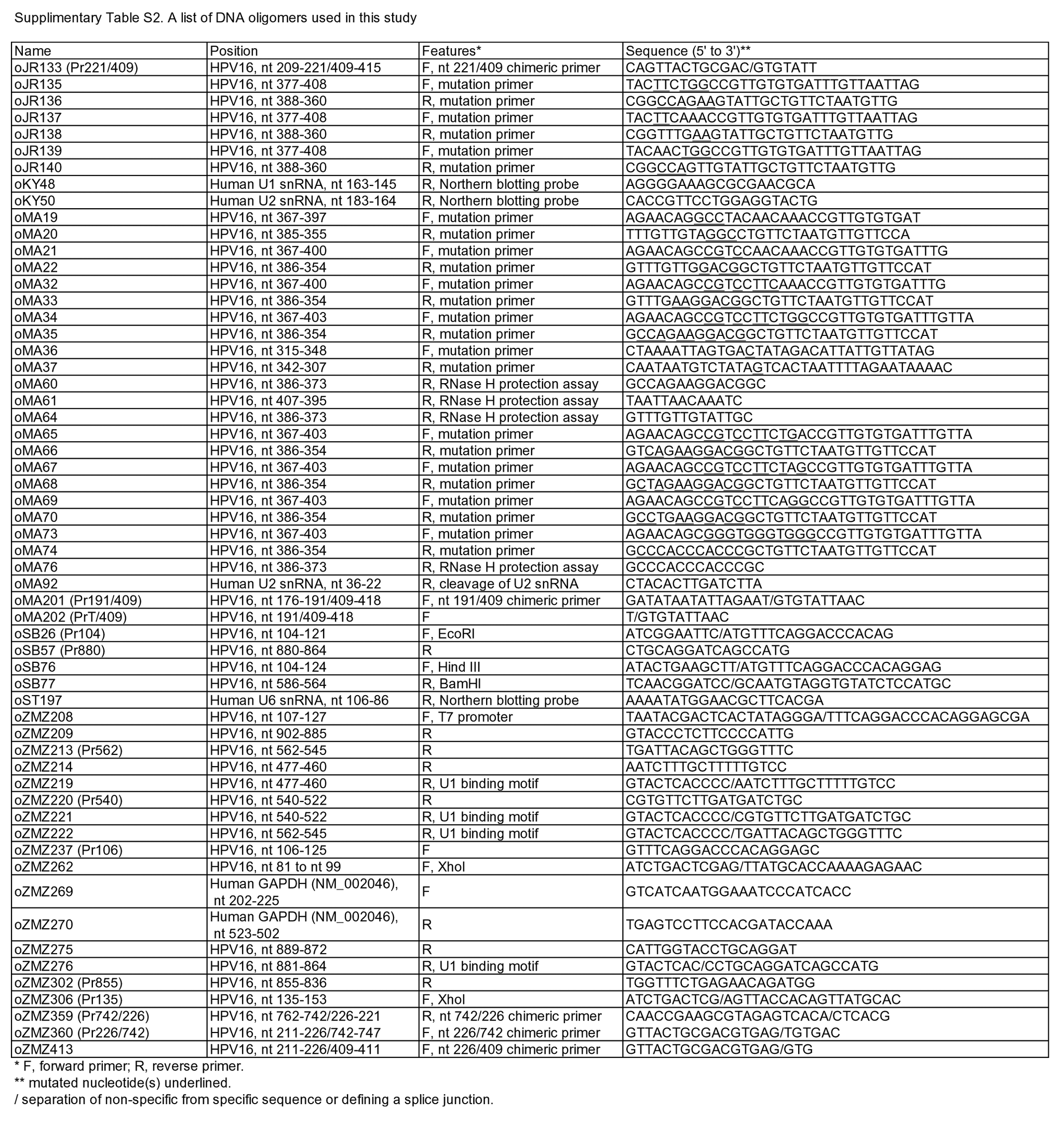

Supplement: Table S2 — A list of DNA oligomers used in this study. (TIF) [file pone.0046412.s005.tif]

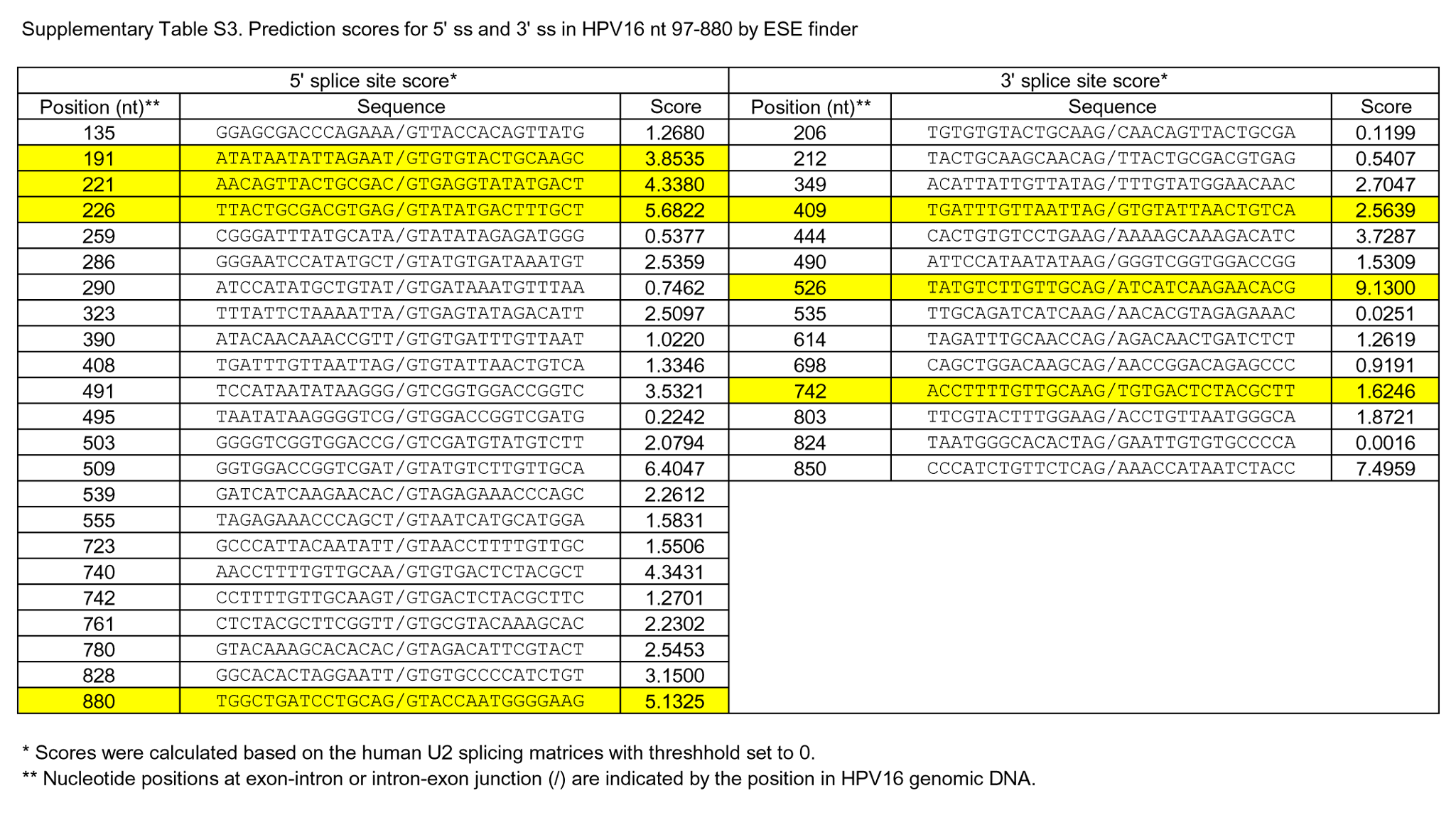

Supplement: Table S3 — Prediction scores for 5′ ss and 3′ ss in HPV16 nt 97–880 by ESE finder. (TIF) [file pone.0046412.s006.tif]

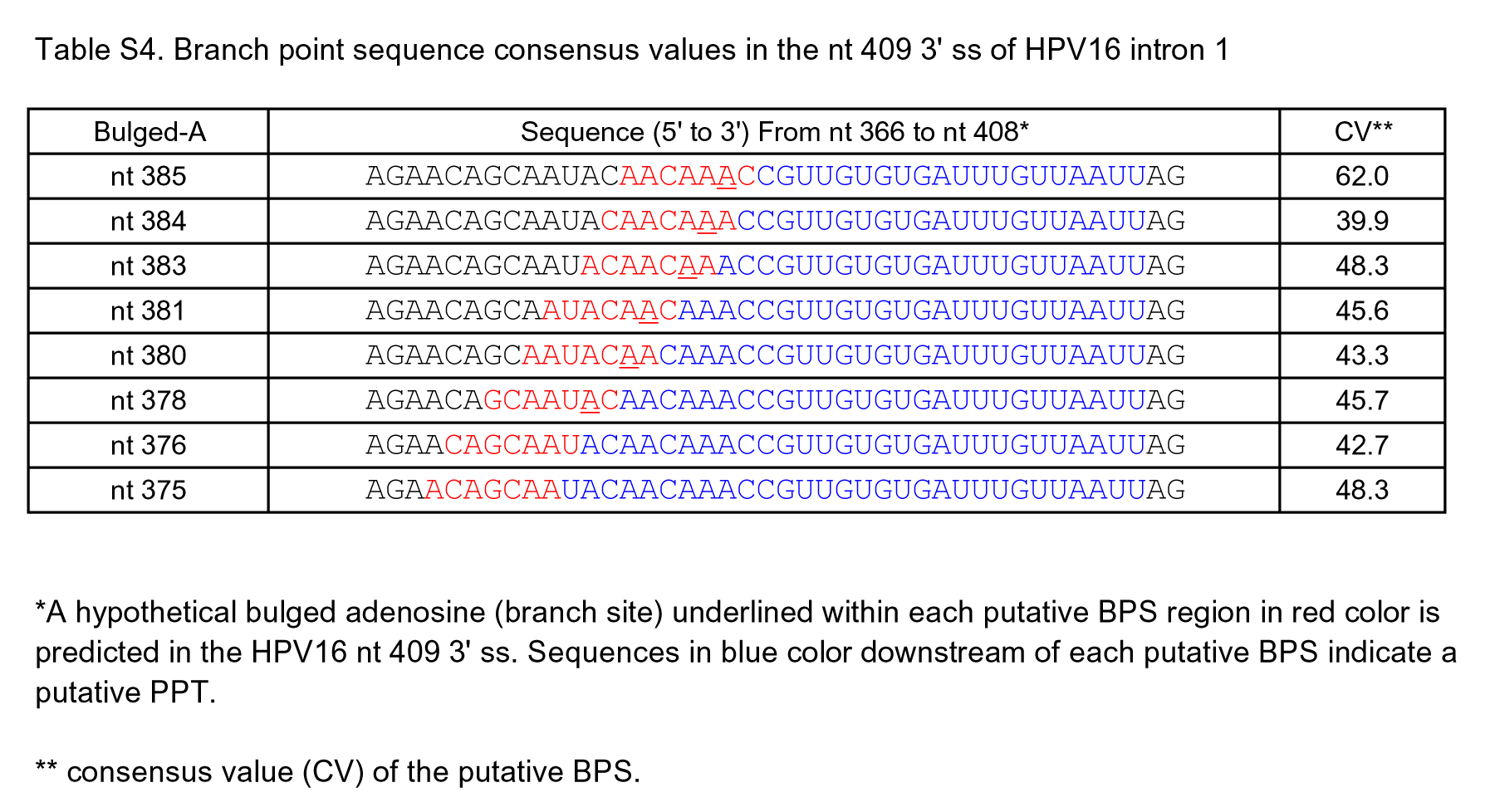

Supplement: Table S4 — Branch point sequence consensus values in the nt 409 3′ ss of HPV16 intron 1. (TIF) [file pone.0046412.s007.tif]
